# Thr55 phosphorylation of p21 by MPK38/MELK ameliorates defects in glucose, lipid, and energy metabolism in diet-induced obese mice

**DOI:** 10.1038/s41419-019-1616-z

**Published:** 2019-05-16

**Authors:** Hyun-A. Seong, Hyunjung Ha

**Affiliations:** Department of Biochemistry, School of Biological Sciences, Chungbuk National University, Cheongju, 28644 Republic of Korea

**Keywords:** Kinases, Obesity

## Abstract

Murine protein serine-threonine kinase 38 (MPK38)/maternal embryonic leucine zipper kinase (MELK), an AMP-activated protein kinase (AMPK)-related kinase, has previously been shown to interact with p53 and to stimulate downstream signaling. p21, a downstream target of p53, is also known to be involved in adipocyte and obesity metabolism. However, little is known about the mechanism by which p21 mediates obesity-associated metabolic adaptation. Here, we identify MPK38 as an interacting partner of p21. p21 and MPK38 interacted through the cyclin-dependent kinase (CDK) binding region of p21 and the C-terminal domain of MPK38. MPK38 potentiated p21-mediated apoptosis and cell cycle arrest in a kinase-dependent manner by inhibiting assembly of CDK2-cyclin E and CDK4-cyclin D complexes *via* induction of CDK2-p21 and CDK4-p21 complex formation and reductions in complex formation between p21 and its negative regulator mouse double minute 2 (MDM2), leading to p21 stabilization. MPK38 phosphorylated p21 at Thr55, stimulating its nuclear translocation, which resulted in greater association of p21 with peroxisome proliferator-activated receptor γ (PPARγ), preventing the PPARγ transactivation required for adipogenesis. Furthermore, restoration of p21 expression by adenoviral delivery in diet-induced obese mice ameliorated obesity-induced metabolic abnormalities in a MPK38 phosphorylation-dependent manner. These results suggest that MPK38 functions as a positive regulator of p21, regulating apoptosis, cell cycle arrest, and metabolism during obesity.

## Introduction

Murine protein serine-threonine kinase 38 (MPK38)/maternal embryonic leucine zipper kinase (MELK), a member of the AMP-activated protein kinase (AMPK)-related kinase family, has been proposed to be involved in the control of a variety of biological processes, including the cell cycle, cell proliferation, apoptosis, signal transduction pathways, tumorigenesis, and metabolism^1,2^. An understanding of its multi-site protein phosphorylation, a common mechanism for regulating protein function and stability, should provide insight into the mechanisms by which MPK38 regulates the activity of its substrates^3^. For example, MPK38 has been shown to phosphorylate Bcl-G, a pro-apoptotic protein, leading to the inhibition of Bcl-G-induced apoptosis^4^. MPK38 phosphorylates ZPR9, a zinc finger protein, and stimulates its nuclear localization, which results in greater B-myb transactivation^5^. Conversely, MPK38 phosphorylates PDK1 at Thr354, thereby inhibiting its activity and function^6^. Furthermore, MPK38 phosphorylates ASK1 (Thr838), p53 (Ser15), and Smads (Ser245 of Smad2, Ser204 of Smad3, Ser343 of Smad4, and Thr96 of Smad7), activating the downstream pathways^2,7,8^. Phosphorylation of serine-threonine kinase receptor-associated protein (STRAP) at Ser188 by MPK38 activates ASK1/TGF‐β/p53 signaling and inactivates PI3K/PDK1 signaling, leading to apoptotic cell death^9^. However, Thr76 phosphorylation of thioredoxin (Trx) by MPK38 inhibits activation of ASK1/TGF‐β/p53 signaling^10^. Further study of such mechanisms should greatly enhance our understanding of the biological roles of MPK38.

p21, a cyclin-dependent kinase (CDK) inhibitor, was initially isolated as a p53-inducible protein and is known to regulate the cell cycle and DNA replication. It has also been implicated in various other biological processes, including apoptosis, cell differentiation, development, tumorigenesis, and metabolism^11,12^. Consistent with its multiple cellular functions, a number of proteins have been found to interact with p21 in cells. In addition to the binding of p21 to cyclin-CDKs and proliferating cell nuclear antigen (PCNA)^13–15^, which leads to the inhibition of cell cycle progression and DNA synthesis, a variety of other proteins have been found to bind to p21; for example, transcription factors^16–19^, pro-apoptotic proteins^20^, protein kinases^21–23^, and many others^24–28^. p21 has also been shown to function as a positive regulator of the cell cycle; for example, p21 overexpression improves cell survival in the face of prostaglandin A2 or p53 overproduction^29,30^. p21 expression is also high in various tumors, particularly in late-stage, aggressive tumors, such as glioblastoma multiforme (GBM)^31–33^. In addition, p21 has been shown to play an important role in adipocyte differentiation: p21 knockdown by RNA interference in 3T3-L1 cells or its ablation in p21^−/−^ mouse embryonic fibroblasts (MEFs) inhibits adipocyte differentiation, suggesting that p21 is a pro-adipogenic factor^34^. By contrast, other studies have shown that mice lacking p21 become obese as a result of adipocyte hyperplasia^35^, which implies that p21 may function as an anti-adipogenic factor. Thus, the precise role of p21 in adipogenesis remains controversial.

In this study, we show that MPK38 phosphorylates p21 at Thr55 and functions as a positive regulator of multiple p21 effects by stabilizing the protein. Moreover, we show that phosphorylated p21 significantly inhibits adipocyte differentiation and ameliorates obesity-induced metabolic abnormalities, implying that MPK38-induced Thr55 phosphorylation of p21 plays a role in the regulation of metabolism in obesity.

## Results

### In vitro and in vivo association of p21 with MPK38

**Fig. 1:**
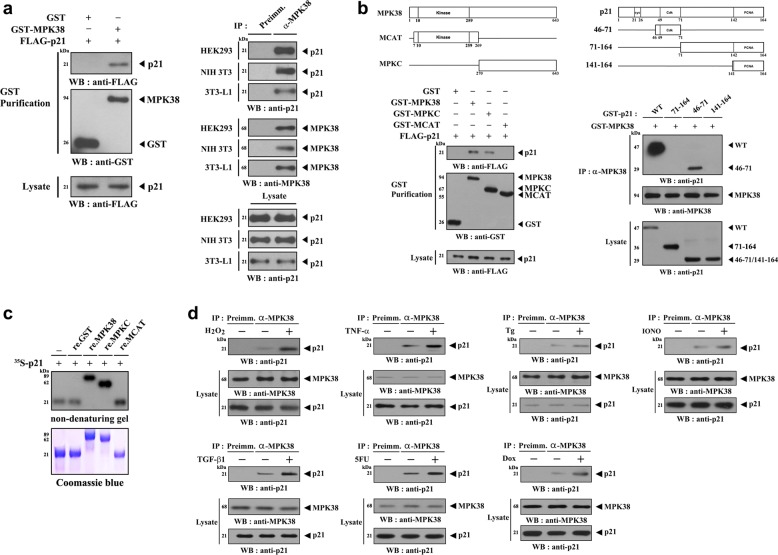
Biochemical characterization of MPK38-p21 interaction. **a** The physical interaction between MPK38 and p21. GST-tagged MPK38 proteins were purified on glutathione-sepharose beads (GST Purification), and the extent of complex formation was measured by immunoblotting with an anti-FLAG antibody in HEK293 cells. Cell lysates (HEK293, NIH 3T3, and 3T3-L1 cells) were immunoprecipitated with either rabbit pre-immune serum (IP: Preimm.) or anti-MPK38 antibody (IP: α-MPK38), followed by immunoblotting with an anti-p21 antibody, to measure the extent of endogenous MPK38-p21 interaction. **b** Mapping of the binding sites involved in MPK38-p21 interaction. Schematic structures of MPK38 and p21 are depicted, and the numbers indicate the amino acid residues at the domain boundaries (upper panels). Cyc, cyclin-binding domain; Cdk, CDK-binding domain; PCNA, PCNA-binding domain. HEK293 cells were transiently transfected with the indicated plasmids, and the extent of MPK38-p21 complex formation was measured by immunoblotting with an anti-FLAG antibody or an anti-p21 antibody. **c** For native PAGE (8%) of the MPK38-p21 complex, recombinant MPK38 (WT, MCAT, or MPKC) was incubated with in vitro-translated ^35^S-labeled wild-type p21 at room temperature for 1 h. Coomassie staining allowed visualization of protein bands. Experiments were performed independently at least three times, with similar results. re., recombinant. **d** Modulation of the MPK38-p21 interaction by various stress stimuli. Cell lysates from HEK293 cells were treated with or without various stress stimuli activating ASK1/TGF-β/p53 signaling pathways, including H_2_O_2_ (2 mM, 30 min), TNF-α (500 ng/ml, 30 min), thapsigargin (Tg: 20 μM, 30 min), ionomycin (IONO: 1 μM, 24 h), TGF-β1 (100 ng/ml, 20 h), 5-fluorouracil (5FU: 0.38 mM, 30 h), and doxorubicin (Dox: 100 ng/ml, 24 h). The extent of the endogenous MPK38-p21 interaction was analyzed by immunoblotting with an anti-p21 antibody

Given that MPK38 stimulates p53 signaling^7^ and regulates the cell cycle^36^, we reasoned that MPK38 might regulate the activity of p21, a p53 target. To investigate this possibility, we first determined whether p21 physically associates with MPK38 using co-transfection experiments. p21 was only detected in the presence of glutathione S-transferase (GST)-tagged MPK38 but not control GST alone (Fig. 1a, left). An endogenous interaction of p21 with MPK38 was also detected in HEK293, NIH 3T3, and 3T3-L1 cells using co-immunoprecipitation experiments (Fig. 1a, right). We then identified the domains involved in the binding of MPK38 to p21. WT MPK38 and MPKC, which contains the carboxy-terminal domain (amino acids 270–643), bound to p21, but MCAT, which contains the N-terminal kinase domain (amino acids 7–269), did not (Fig. 1b, left). By contrast, MPK38 interacted with the p21 deletion construct 46–71 (amino acids 46–71), which contains the CDK-binding site, but not with the 71–164 construct (amino acids 71–164) or the 141–164 construct (amino acids 141–164), which contains the PCNA binding site, in HEK293 cells (Fig. 1b, right). These results imply that the interaction is mediated via the CDK-binding region of p21 and the C-terminal domain of MPK38 in cells.

Next, to examine whether the interaction was direct, we performed non-denaturing polyacrylamide gel electrophoresis (PAGE) using GST-tagged recombinant MPK38 (WT, MPKC, or MCAT) and in vitro-translated ^35^S-labeled p21. A shift in the mobility of radiolabeled p21 was only detected in the presence of recombinant WT MPK38 or MPKC but not MCAT or control GST alone, revealing that p21 and MPK38 directly interact (Fig. 1c). In addition, ASK1/TGF-β/p53 signals increased the formation of p21-MPK38 endogenous complexes (Fig. 1d), suggesting a critical role for stress signals in the regulation of the p21-MPK38 interaction.

### MPK38 phosphorylates p21 at Thr55 and stimulates its nuclear translocation

**Fig. 2:**
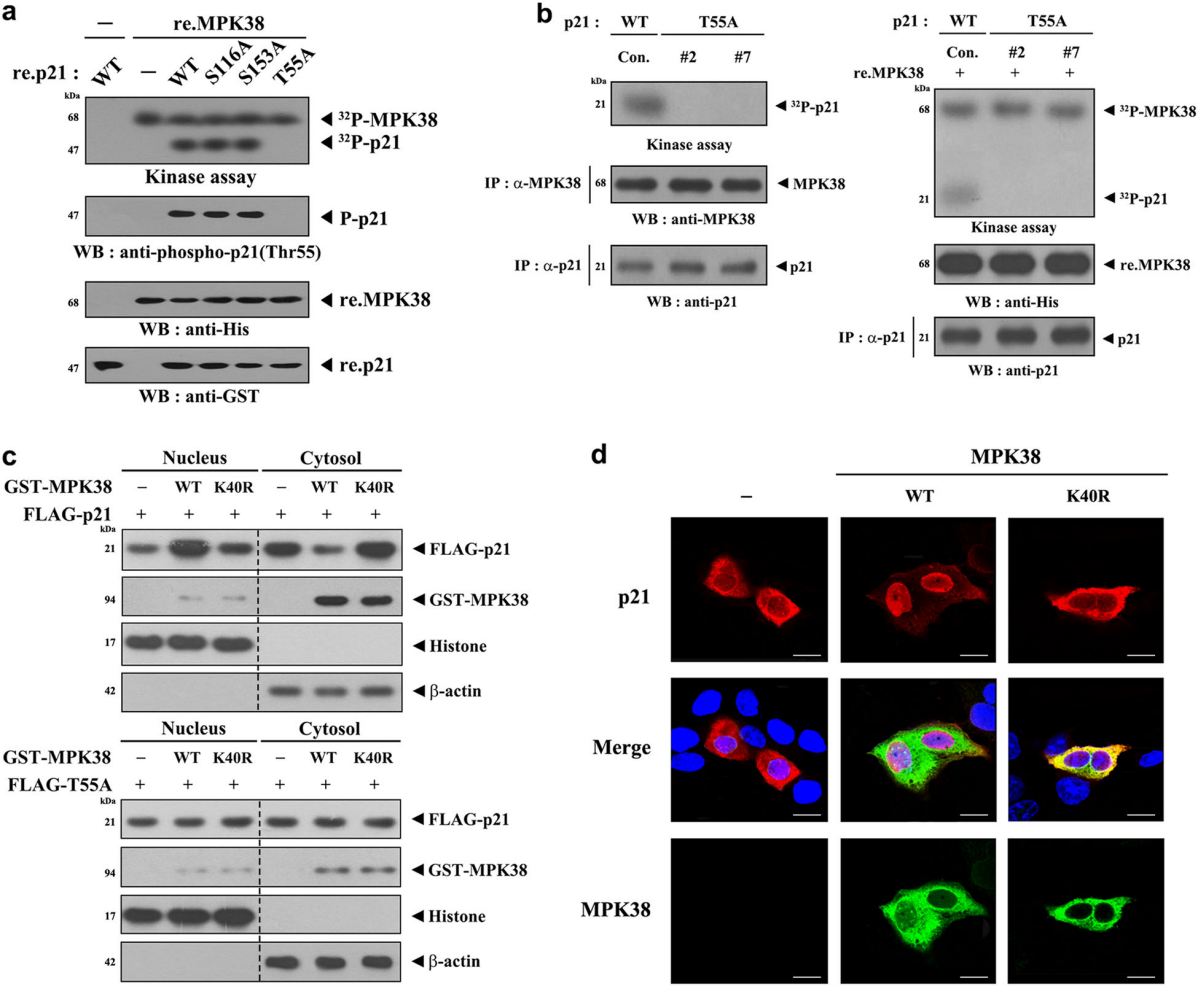
Thr55 phosphorylation by MPK38 and nuclear translocation of p21. **a** In vitro kinase assays were carried out using recombinant MPK38 protein and wild-type (WT) p21 or a p21 substitution mutant (S116A, S153A, or T55A) as the substrate in the presence of 5 μCi of [γ-^32^P]-ATP to identify the MPK38 phosphorylation site on p21. ^32^P, ^32^P incorporation; P, phosphorylated; re., recombinant; WB, western blot. **b** Cell lysates of wild-type (control) and CRISPR/Cas9 p21 (T55A) KI clones (#2 and #7) were immunoprecipitated with an anti-MPK38 antibody or an anti-p21 antibody. MPK38 kinase assay was carried out using p21 immunoprecipitate as the substrate in the presence of MPK38 immunoprecipitate (left). Recombinant MPK38 proteins were also used instead of the MPK38 immunoprecipitate in the MPK38 kinase assay (right). IP, immunoprecipitation; KI, knock-in. **c** Effect of MPK38 on the subcellular localization of p21. HEK293 cells transfected with the indicated expression vectors were lysed, and nuclear and cytosolic fractions were prepared for immunoblot analyses. p21 protein levels were assessed by immunoblotting with an anti-FLAG antibody (top panels). **d** 3T3-L1 cells transfected with the indicated plasmids were immunostained with anti-rabbit primary p21 and anti-mouse primary MPK38 antibodies, detected respectively with Alexa Fluor-594 anti-rabbit secondary antibody (red fluorescence, for p21) or Alexa Fluor-488 anti-mouse secondary antibody (green fluorescence, for MPK38). The 4′,6-diamidino-2-phenylindole (DAPI) stained nucleus has blue fluorescence. Scale bar, 10 μm

To determine whether p21 can be a direct substrate for MPK38, recombinant MPK38 protein was incubated with [γ-^32^P]ATP and the phosphorylation of recombinant p21 protein was measured. p21 phosphorylation was identified in the presence of MPK38 (Fig. 2a, 3rd lane). To define the MPK38 phosphorylation sites on p21, we searched for consensus MPK38 phosphorylation sites^37^ in p21 and performed in vitro kinase assays using three selected substitution mutants (S116A, S153A, and T55A) of p21. The T55A mutation, but not the S116A and S153A mutations, completely abolished MPK38-mediated phosphorylation (Fig. 2a, 6th lane), indicating that Thr55 of p21 is a potential MPK38 phosphorylation site. In vivo phosphorylation of p21 by MPK38 was confirmed using p21 knock-in (T55A) 3T3-L1 cells generated using the CRISPR/Cas9 system (Fig. 2b).

We then analyzed the effect of MPK38 on the subcellular localization of p21 because its localization is affected by phosphorylation events^38^. Although p21 was mainly expressed in the cytoplasm in 3T3-L1 cells, it translocated to the nucleus in the presence of WT MPK38, but not kinase-dead (K40R) MPK38 (Fig. 2c, upper). This implies that Thr55 phosphorylation of p21 is an important trigger for the nuclear translocation of p21. However, we did not observe any change in the intracellular distribution of the T55A mutant under the same conditions (Fig. 2c, lower). MPK38-induced nuclear localization of p21 was also demonstrated using immunofluorescence microscopy (Fig. 2d). These results suggest that Thr55 phosphorylation of p21 triggers the translocation of p21 from the cytoplasm to the nucleus.

### MPK38 reduces the assembly of both the CDK2-cyclin E and CDK4-cyclin D complexes

**Fig. 3:**
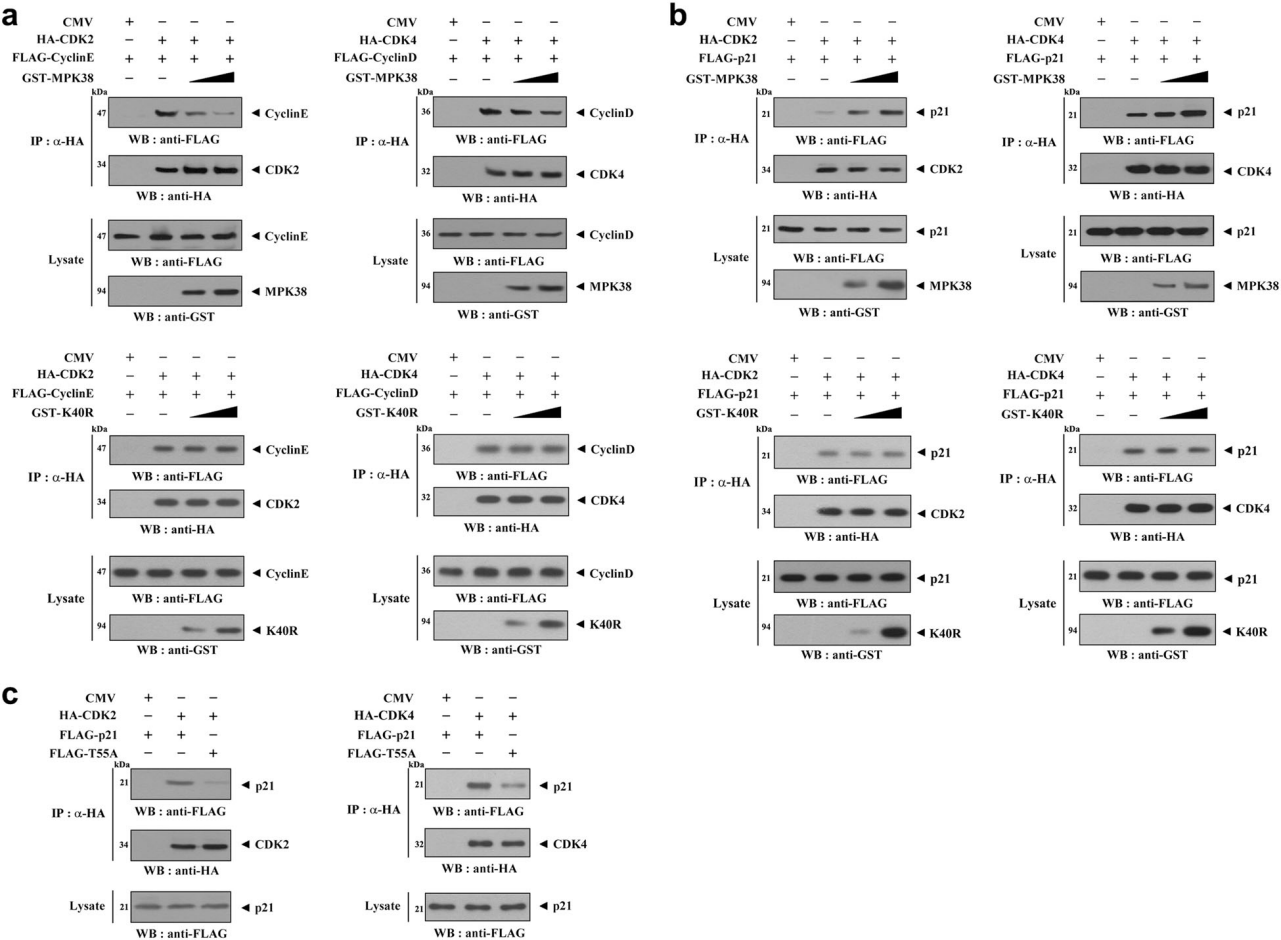
Inhibition of the assembly of the CDK2-cyclin E and CDK4-cyclin D complex by MPK38. To assess the extent of CDK2-cyclin E or CDK4-cyclin D interactions (**a**) and CDK2-p21 or CDK4-p21 interactions (**b**, **c**), HEK293 cells were transiently transfected with the indicated plasmids for HA-CDK2/4, FLAG-cyclin E/D, and FLAG-p21/T55A in the presence or absence of WT or a kinase-dead mutant (K40R) of MPK38. After 48 h, cell lysates were immunoprecipitated with anti-HA antibody (IP: α-HA), and the extent of complex formation was measured by immunoblotting with an anti-FLAG antibody. Whole-cell lysates were probed with anti-FLAG and anti-GST antibodies (**a**, **b**), or an anti-FLAG antibody (**c**), to quantify expression following transfection

p21 has been shown to inhibit the assembly and activation of both the CDK2-cyclin E and CDK4-cyclin D complexes^15^. To determine whether MPK38 affects the formation of these complexes, we analyzed their assembly in the presence of MPK38. MPK38 reduced the assembly of both complexes in a dose-dependent manner, whereas K40R MPK38 had no such effect (Fig. 3a). Also, MPK38 increased the binding of p21 to CDK2 or CDK4 in a kinase-dependent manner (Fig. 3b), suggesting that phosphorylated p21 is capable of interacting with and inhibiting CDK2 or CDK4, and possibly inhibiting cell cycle progression. To confirm this, the association of p21 with CDK2 or CDK4 was analyzed using both WT and T55A p21, and indeed these complexes were more common in the presence of WT p21 than in the presence of the T55A mutant (Fig. 3c). These data suggest that MPK38 interacts with and phosphorylates p21, resulting in the induction of CDK2-p21 and CDK4-p21 complex formation, and subsequent inhibition of the assembly of CDK2-cyclin E and CDK4-cyclin D complexes.

### MPK38 stimulates p21 promoter activity, and p21-mediated apoptosis and growth arrest, in a kinase-dependent manner

**Fig. 4:**
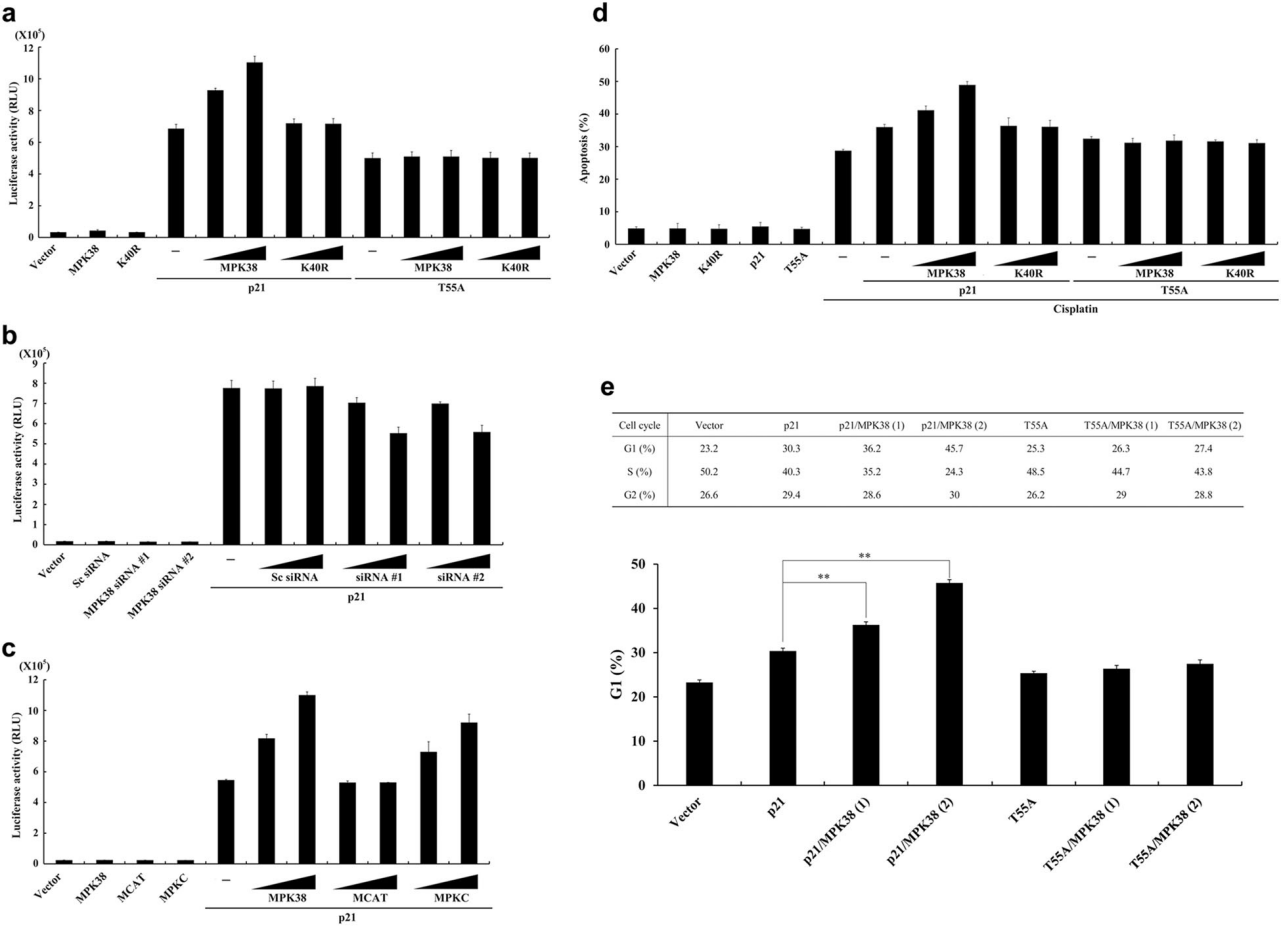
Stimulation of p21 promoter activity, and p21-mediated apoptosis and growth arrest, by MPK38. **a**–**c** Stimulation of p21 promoter activity by MPK38. HCT116 cells expressing wild-type p53, required for p53/p300-induced p21 promoter activity, were transiently transfected with WWP-Luc plasmid (0.2 μg) and two different amounts of MPK38 (WT and K40R: 0.4 or 0.8 μg), MCAT and MPKC (0.4 or 0.8 μg), MPK38-specific siRNAs (#1 or #2: 100 or 200 nM)^6^, and scrambled siRNA (Sc: 100 or 200 nM)^6^, as indicated. The data shown are means (± SEM) of three independent experiments. **d** Stimulation of p21-mediated apoptosis by MPK38. MCF7 cells expressing wild-type p53, an important mediator of cisplatin-induced apoptosis, were transiently transfected with p21 (WT and T55A: 0.4 μg) and MPK38 (WT and K40R: 0.3 or 0.6 μg) in the presence or absence of cisplatin (1 μg/ml). GFP expression system was used to detect cell death^2^. **e** Stimulation of p21-mediated growth arrest by MPK38. p53-null H1299 cells were transfected with p21 (WT and T55A: 0.4 μg) and MPK38 (WT: 0.3 or 0.6 μg), and fixed for FACS analysis 40 h after transfection and 16 h after nocodazole treatment. The data are also shown in the graph where y-axis indicates the percentage of cells in G1 phase. **p < 0.01 compared to control expressing p21. MPK38 (1) and (2) indicate the use of 0.3 μg and 0.6 μg of MPK38, respectively

To explore the functional significance of the p21-MPK38 interaction, we first performed reporter assays to quantify the effect of MPK38 on p53/p300-induced p21 promoter activity because p21 increased the formation of complexes of p53 and its coactivator, p300, in an MPK38 phosphorylation-dependent manner (Fig. S1). We found that MPK38 increased p21 promoter activity in a kinase- and dose-dependent manner (Fig. 4a). This suggests that the kinase activity of MPK38 is important for the regulation of p21 function. To confirm this positive influence of MPK38 on p21 promoter activity, we carried out a knockdown experiment using MPK38-specific siRNAs and found that down-regulation of endogenous MPK38 reduced p21 promoter activity in a dose-dependent manner (Fig. 4b). In a separate experiment using two MPK38 deletion constructs (MCAT and MPKC), MPKC dose-dependently stimulated p21 promoter activity (Fig. 4c). However, this effect was not observed in the presence of MCAT, which fails to bind to p21 (Fig. 1b). This indicates that both direct interaction and phosphorylation of p21 by MPK38 play important roles in the upregulation of p21 promoter activity. Consistent with this, MPK38 increased p21-mediated apoptosis in a kinase-dependent manner, but this stimulatory effect did not occur in the presence of the T55A mutation (Fig. 4d).

Next, to determine whether MPK38 influences p21-induced cell cycle arrest, we performed flow cytometry analysis using H1299 cells. As shown in Fig. 4e, a much larger number of cells (~46% vs. ~30%) was found to be in G1 phase after co-transfection with MPK38 and p21 than in controls transfected with p21 alone. However, this effect was barely detectable in the presence of the T55A mutant (~27% vs. ~25%). These findings suggest that MPK38 stimulates p21-mediated phenomena, such as apoptosis and cell cycle arrest, in a kinase-dependent manner.

### MPK38 potentiates p21-mediated inhibition of adipocyte differentiation

**Fig. 5:**
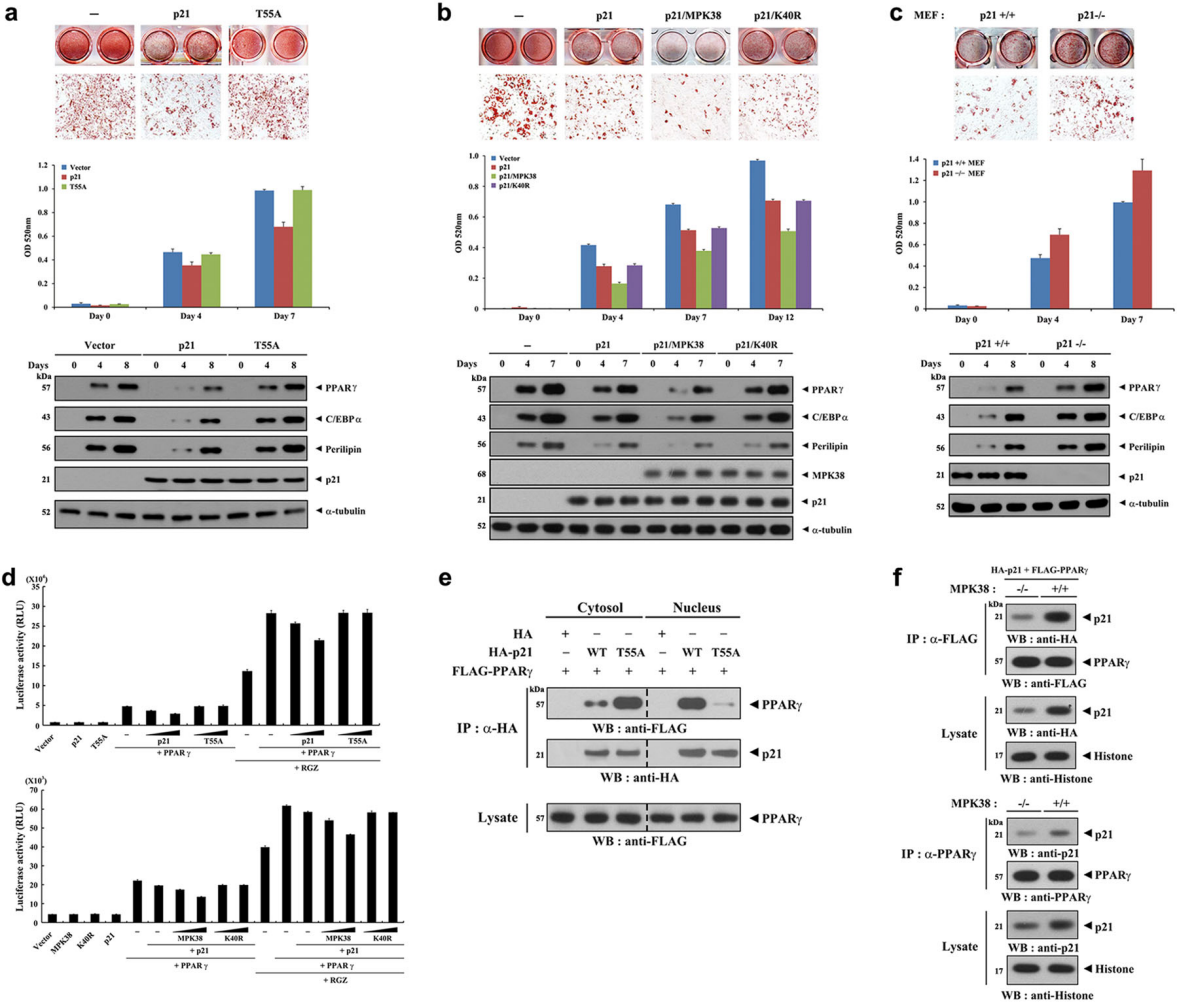
Inhibition of adipocyte differentiation by Thr55 phosphorylation of p21. **a**–**c** Effect of Thr55 phosphorylation of p21 on adipocyte differentiation. Oil Red O staining (top), measurements of absorbance at 520 nm (middle), and immunoblot analyses (bottom) were performed using 3T3-L1 preadipocytes transfected with the indicated plasmids (**a**, **b**) or MEF cells expressing p21 (+/+) or not (−/−) (**c**) on the indicated number of days after the start of differentiation. Oil Red O staining of cells 10−14 days after the start of differentiation is shown. **d** Effect of Thr55 phosphorylation of p21 on PPARγ-mediated transactivation. 3T3-L1 cells were transfected with 0.2 μg PPARγ luciferase plasmid, two different amounts of p21 (WT and T55A: 0.1 or 0.2 μg) and MPK38 (WT and K40R: 0.1 or 0.2 μg), p21 (0.2 μg), or PPARγ (0.3 μg) in the presence or absence of RGZ (10 μM). The data shown are means (± SEM) of three independent experiments. **e** Effect of Thr55 phosphorylation on the extent of p21-PPARγ complex formation in the nucleus. HEK293 cells transiently transfected with the indicated expression vectors were lysed, and nuclear and cytosolic fractions were prepared for immunoblot analysis. The extent of p21-PPARγ complex formation was assessed by immunoblotting with an anti-FLAG antibody (top panel). **f** Quantification of exogenous (upper) and endogenous (lower) p21-PPARγ complex formation using MEF cells expressing MPK38 (+/+) or not (−/−)

p21 is known to be a critical regulator of adipocyte differentiation, which involves a sequence of cell cycle arrests^34,35^. To determine the effects of p21 on the differentiation of 3T3-L1 adipocytes, WT and T55A p21 were overexpressed before the induction of their differentiation. The expression of WT p21 suppressed adipocyte differentiation, as assessed using Oil Red O staining. However, no such effect was observed in the presence of the T55A mutant (Fig. 5a, top and middle). The expression of WT p21 was accompanied by lower expression of PPARγ and CCAAT-enhancer-binding protein α (C/EBPα), which are involved in adipocyte differentiation, along with lower expression of the mature adipocyte marker perilipin (Fig. 5a, bottom). In addition, the mRNA expression of other key adipogenic genes, such as fatty acid binding protein 4 (*Fabp4*) and adiponectin (*Adipoq*), was also lower in 3T3-L1 cells expressing WT p21 than in control cells expressing an empty vector alone (Fig. S2). However, these differences in expression were barely detectable in cells expressing T55A p21. The importance of MPK38-mediated Thr55 phosphorylation of p21 in the inhibition of adipocyte differentiation was also verified using 3T3-L1 cells transfected with p21 alone, or co-transfected with p21 and MPK38, as assessed using Oil Red O staining and immunoblot analysis (Fig. 5b). This finding was further confirmed using MEFs from p21-null mice because p21^−/−^ MEFs displayed a greater degree of adipocyte differentiation than control p21^+/+^ MEFs (Fig. 5c).

To elucidate how p21 inhibits adipogenesis, we assessed the transcriptional activity of PPARγ, a key transcription factor in adipogenesis^39–41^, using a luciferase assay with p21 in the presence of rosiglitazone (RGZ), a PPARγ agonist. We found that WT p21 suppressed the transcriptional activity of PPARγ in a dose-dependent manner, whereas the T55A mutant, which is unable to translocate to the nucleus (see Fig. 2c), did not have a suppressive effect under the same conditions (Fig. 5d, upper). The importance of Thr55 phosphorylation of p21 in the suppression of PPARγ transcriptional activity was also demonstrated using a reporter assay with MPK38 in the presence of p21 (Fig. 5d, lower). These findings indicate that nuclear translocation of p21 is important for the negative regulation of PPARγ. Indeed, the WT p21 strongly bound to PPARγ in the nucleus, but not in the cytoplasm (Fig. 5e). Consistent with this, MPK38^+/+^ MEFs displayed greater exogenous and endogenous complex formation between p21 and PPARγ in the nucleus than MPK38^−/−^ MEFs (Fig. 5f), suggesting that p21 may function as a repressor of PPARγ. These results imply that MPK38 negatively regulates adipogenesis by promoting the association of p21 with PPARγ in the nucleus, thereby preventing the transcriptional activity of PPARγ.

### Thr55 phosphorylation of p21 contributes to the amelioration of adiposity and impaired glucose and energy metabolism in diet-induced obese mice

**Fig. 6:**
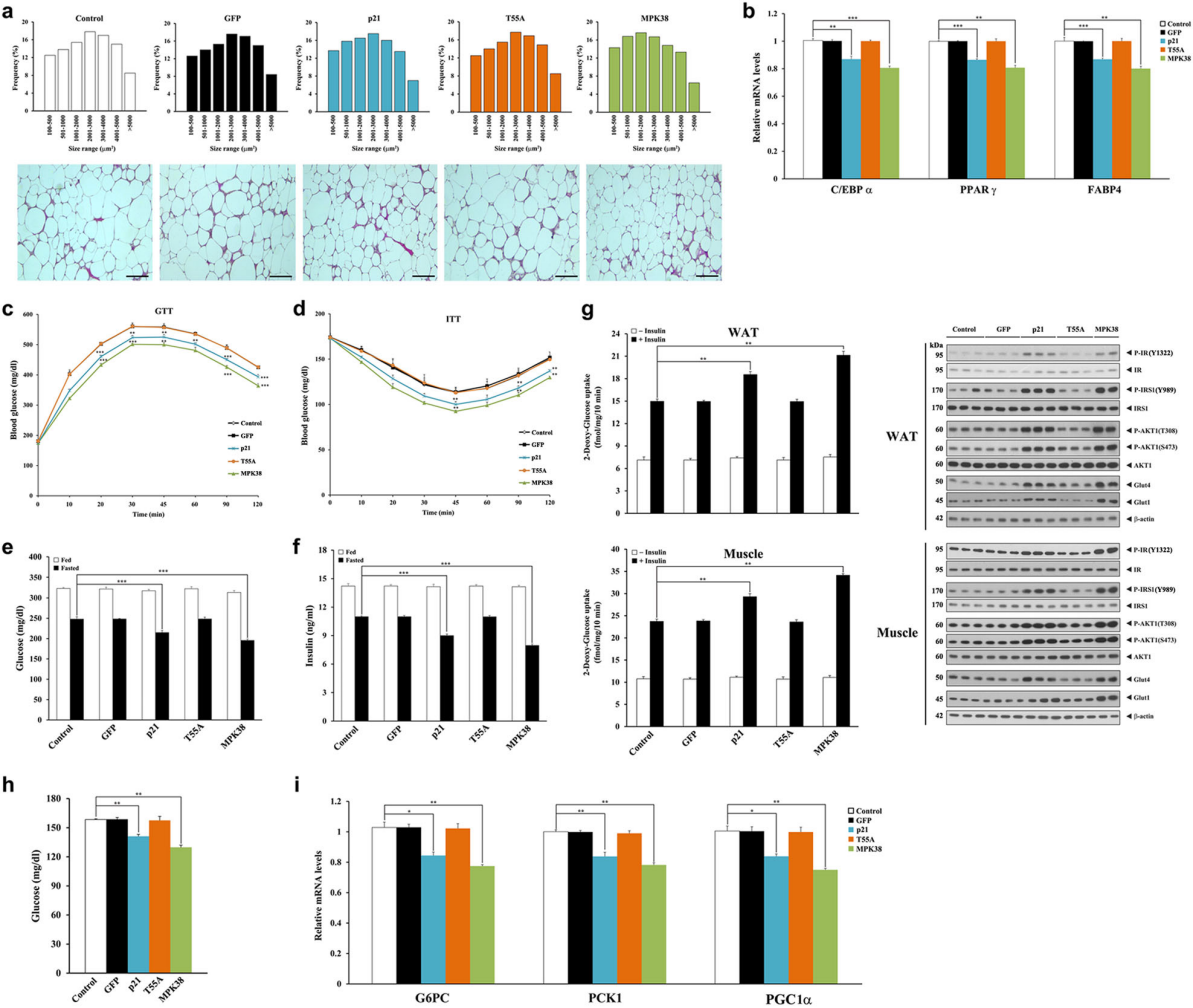
Amelioration of adiposity and impaired glucose metabolism following Thr55 phosphorylation of p21 in diet-induced obese mice. **a** Size distribution analysis of adipocytes (upper) and paraffin-embedded hematoxylin and eosin sections of epididymal WAT (lower) from HFD-fed mice (5–7 days after adenoviral infection). Ad-MPK38 was used as a positive control. Scale bar, 100 μm. **b** Relative mRNA expression of adipogenic genes. The fold change in expression relative to control is presented. **c**, **d** GTTs and ITTs were performed as described in the “Materials and methods”. **p < 0.01, ***p < 0.001 compared to control, determined by two-way ANOVA. **e**, **f** Fed and fasting (10 h) blood glucose and insulin levels. ***p < 0.001 compared to fasted controls, determined by two-way ANOVA. **g** 2-deoxy-glucose uptake in the presence or absence of human insulin (10 mU/ml) (left). **p < 0.01 compared to controls treated with insulin, determined by two-way ANOVA. IRS-PI3K signaling was measured by immunoblotting (right) after injection of insulin into the caudal vena cava (*n* = 3 per group)^42^. **h**, **i** Blood glucose (**h**) and relative mRNA expression of hepatic gluconeogenic genes (glucose-6-phosphatase catalytic subunit (*G6pc*), phosphoenolpyruvate carboxykinase-1 (*Pck1*), and peroxisome proliferator-activated receptor γ coactivator 1α (*Pgc1α*)) (**i**). *n* = 6 per group (from **a** to **i** except **g**), *p < 0.05, **p < 0.01, ***p < 0.001 compared to control (**b**, **h**, **i**). The experiments were performed in duplicate and repeated 3 times with similar results (**b**, **i**)

Our previous studies have shown that obesity is inversely associated with MPK38 and p21 expression in mice^42^. Therefore, an adenoviral delivery system was employed to investigate whether the restoration of p21 expression would ameliorate the obesity-induced metabolic abnormalities in male C57BL/6 mice fed an HFD. The adenoviral delivery of p21 (Ad-p21) reduced the number of large hypertrophic adipocytes (Fig. 6a) and the mRNA expression of key adipogenic regulators (Fig. 6b), including C/EBPα, PPARγ, and FABP4, when compared with uninfected or Ad-GFP-infected HFD-fed C57BL/6 mice. However, Ad-T55A infection had no such effect, implying a significant role of Thr55 phosphorylation of p21 in the regulation of adipocyte metabolism and obesity.

Ad-p21 infection improved glucose tolerance and insulin sensitivity in HFD-fed C57BL/6 mice (Fig. 6c, d). In parallel, Ad-p21 infection caused lower circulating glucose and insulin concentrations under fasting conditions (Fig. 6e, f) and stimulated in vitro insulin-stimulated 2-deoxy-glucose uptake into white adipose tissue (WAT) and muscle (Fig. 6g, left). Ad-p21 infection also increased activation of the insulin receptor substrate (IRS)-phosphoinositide 3-kinase (PI3K) pathway, which stimulates glucose uptake (Fig. 6g, right). Ad-p21 infection caused considerably lower levels of blood glucose (Fig. 6h) and mRNA expression of gluconeogenic genes in liver (Fig. 6i). However, none of these effects were observed after Ad-T55A infection. Such beneficial effects of the adenoviral delivery of p21 were also observed in p21-deficient obese mice fed an HFD (Fig. S3). Regarding energy metabolism, the HFD-fed mice infected with Ad-p21 had increased O_2_ consumption and CO_2_ production (Fig. S4a, b) and exhibited a higher rate of energy expenditure, which was associated with a change in locomotor activity (Fig. S4d, g). Ad-p21 infection altered energy substrate usage, as evidenced by a higher level of respiratory exchange ratio (RER) (Fig. S4c). However, Ad-p21 infection did not affect food and water intake (Fig. S4e, f). All these results indicate that p21 ameliorates defects in adipocyte and glucose and energy metabolism in a MPK38 phosphorylation-dependent manner in diet-induced obese mice.

### Thr55 phosphorylation of p21 contributes to the amelioration of abnormal lipid metabolism and inflammation in diet-induced obese mice

**Fig. 7:**
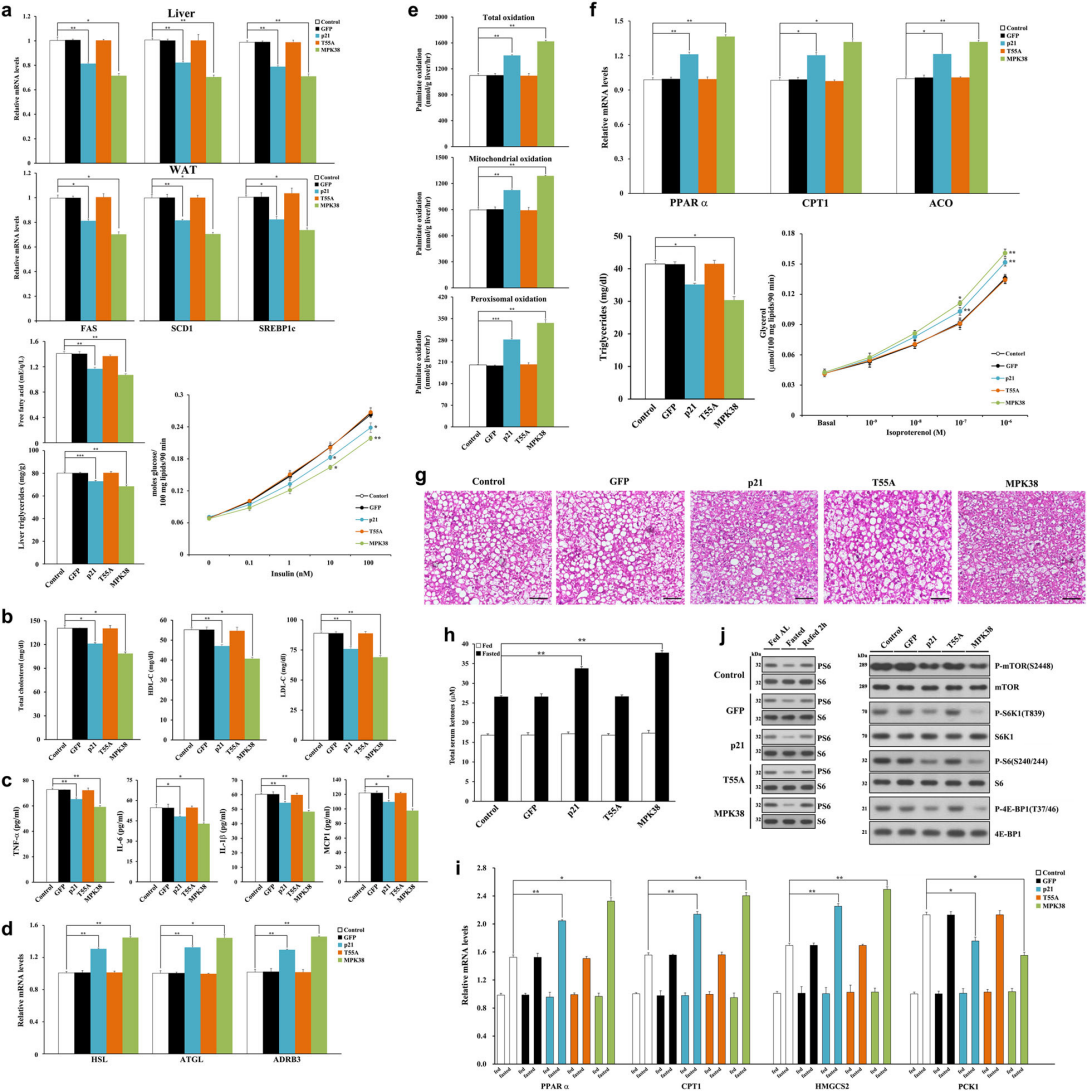
Amelioration of abnormal lipid metabolism and inflammation following Thr55 phosphorylation of p21 in diet-induced obese mice. **a**–**d** Relative mRNA expression of pro-lipogenic (fatty acid synthase (*Fas*), sterol CoA desaturase 1 (*Scd1*), and sterol regulatory element-binding transcription factor 1c (*Srebp1c*)) (**a**) and lipolytic (hormone-sensitive lipase (*Hsl*), adipose triglyceride lipase (*Atgl*), and beta-3 adrenergic receptor (*Adrb3*)) (**d**) genes in liver and epididymal WAT, serum free fatty acid concentration (**a**), liver triglyceride content (**a**), lipogenic capacity of hepatocytes (**a**), serum concentrations of total cholesterol (**b**), HDL-C (**b**), LDL-C (**b**), and proinflammatory proteins (tumor necrosis factor-α (TNF-α), IL-6, IL-1β, and monocyte chemoattractant protein 1 (MCP1)) (**c**). **e**, **f** Measurement of β-oxidation using ^14^C-labeled palmitate in liver (**e**), relative mRNA expression of fatty acid oxidative genes (*Pparα*, carnitine palmitoyltransferase 1 (*Cpt1*), and acyl-CoA oxidase (*Aco*)) in epididymal WAT (**f**), circulating triglyceride concentrations (**f**), and isoproterenol-stimulated lipolysis in primary adipocytes (**f**). **g** Representative images of paraffin-embedded liver sections stained with hematoxylin and eosin. Scale bar, 100 μm. **h**, **i** Total ketone body concentration in fed and fasted (18 h) serum (**h**) and relative mRNA expression of genes involved in ketogenesis (*Pparα*, *Cpt1*, and 3-hydroxy-3-methylglutaryl-CoA synthase 2 (*Hmgcs2*)) in liver (**i**). Phosphoenolpyruvate carboxykinase 1 (*Pck1*), which is not a target of PPARα, was used as a non-specific control. *p < 0.05, **p < 0.01 compared to the fasted control, determined by two-way ANOVA. **j** Phospho-S6 Ser240/244 levels of liver lysates from uninfected and infected HFD-fed mice fed *ad libitum*, 18 h fasted, or 2 h refed (left). Hepatic mTORC1 signaling was also analyzed by immunoblotting (right). *n* = 6 per group (**a**–**j**), *p < 0.05, **p < 0.01, ***p < 0.001 compared to control (**a**–**f**). The qPCR experiments were performed in duplicate and repeated 3 times with similar results (**a**, **d**, **f**, **i**)

We next determined whether p21 regulates the expression of lipogenic genes in HFD-fed C57BL/6 mice. Consistent with the lower circulating free fatty acid (FFA) concentration, Ad-p21 infection reduced the mRNA expression of lipogenic genes in liver and WAT (Fig. 7a). Ad-p21 infection reduced lipogenesis and liver triglyceride content, as well as circulating total cholesterol, high-density lipoprotein (HDL)-cholesterol, and low-density lipoprotein (LDL)-cholesterol concentrations (Fig. 7a, b). We also quantified the expression of proinflammatory genes in serum because cholesterol and fatty acids are involved in inflammatory pathways^43^. Ad-p21 infection considerably reduced the serum concentrations of proinflammatory proteins (Fig. 7c) and the mRNA expression of proinflammatory genes in WAT (Fig. S5), but Ad-T55A infection did not have these effects.

Ad-p21 infection increased the mRNA expression of lipolytic genes in WAT and the hepatic fatty acid utilization by enhancing the mitochondrial and peroxisomal pathways of fatty acid oxidation (Fig. 7d, e). Ad-p21 infection resulted in higher expression of key genes involved in fatty acid oxidation in WAT as well as lower serum triglyceride concentration and greater isoproterenol-stimulated lipolytic activity (Fig. 7f). Consistent with lower liver triglyceride, Ad-p21 infection decreased substantially liver lipid accumulation (Fig. 7a, g). However, none of these effects were observed after Ad-T55A infection. All these data are consistent with Thr55 phosphorylation of p21 upregulating lipid metabolism.

We next investigated whether p21 regulates ketogenesis in the liver. Ad-p21 infection stimulated ketone body production (Fig. 7h) and ketogenic gene expression (Fig. 7i) in response to fasting. Previous studies have implicated ketogenesis in the modulation of cell signaling pathways^44^, and indeed Ad-p21 infection caused lower levels of phospho-S6 Ser240/244 in response to fasting (Fig. 7j, left). Consistent with this, Ad-p21 infection downregulated mechanistic target of rapamycin (mTORC)1 signaling, and this demonstrated a reciprocal trend to ketogenesis (Fig. 7j, right). Similar effects were identified in p21-deficient obese mice fed an HFD (Fig. S6). These results suggest that p21 can ameliorate the hyperlipidemic phenotype of HFD-fed mice in a MPK38 phosphorylation-dependent manner.

### MPK38 increases p21 stability

**Fig. 8:**
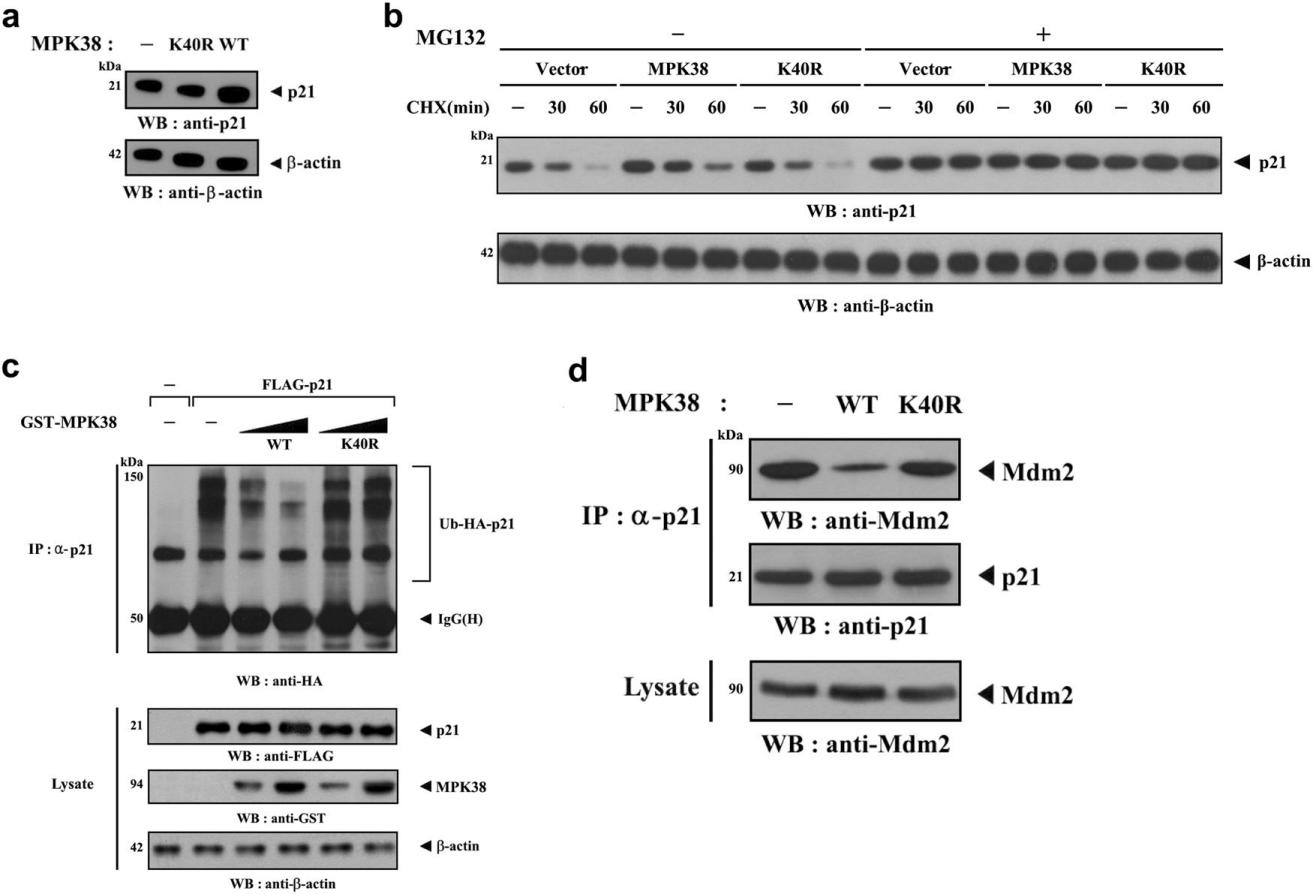
Stabilization of p21 by MPK38. **a**, **b** p21 protein levels were evaluated by immunoblotting with an anti‐p21 antibody. HEK293 cells transfected with vectors encoding MPK38 (WT or K40R) or empty vector (vector) were treated with or without MG132 (10 μM) in the presence of cycloheximide (CHX, 20 μg/ml) for the indicated time intervals. **c** The ubiquitination of endogenous p21 was assessed in HEK293 cells transfected with increasing amounts of WT and K40R MPK38, HA-tagged ubiquitin (Ub), and FLAG-tagged p21 as indicated. **d** HEK293 cells were transfected with vectors encoding MPK38 (WT or K40R), and the extent of endogenous p21–Mdm2 complex formation was measured by immunoblot analysis using an anti-Mdm2 antibody

The transcriptional activation and post-translational modifications play important roles in p21 degradation, although the precise mechanism leading to protein degradation is still controversial^45–47^. To determine whether the high expression of p21 in MPK38-transfected cells (Fig. 8a) is due to protein stabilization, p21 stability was first assessed by immunoblot analysis. p21 was degraded after cycloheximide treatment in either untransfected or K40R MPK38-transfected cells. By contrast, p21 was stabilized in cells transfected with WT MPK38 (Fig. 8b). Moreover, treatment with both cycloheximide and MG132, a proteasomal inhibitor, led to greater stability of p21 than in HEK293 cells that were not treated with MG132, implying an important role for the proteasome pathway in p21 degradation (Fig. 8b). We next analyzed the effect of MPK38 on p21 ubiquitination and found that MPK38 dose-dependently reduced the ubiquitination of p21 in a kinase-dependent manner (Fig. 8c). We also determined whether the regulation of p21 stability by MPK38 is dependent on the documented interaction between p21 and Mdm2^48^, and found that MPK38 markedly reduced endogenous p21–Mdm2 complex formation in a kinase-dependent manner (Fig. 8d). These findings suggest that MPK38 promotes p21 stability through Thr55 phosphorylation.

## Discussion

In this study, we explored the role of MPK38, also known as MELK, on p21 activity and function, and found that MPK38 positively regulates p21-mediated apoptosis, cell cycle arrest, and metabolic regulation by phosphorylating p21 at Thr55. Together with the previous finding that MPK38 activates p53 signaling through direct phosphorylation of p53 at Ser15^7^, our current results indicate that MPK38 stimulates the activity of p21 through both p53-dependent and -independent mechanisms. In order to elucidate the regulatory mechanism of MPK38 on p21 activity, we investigated whether the physical interaction between the two and the phosphorylation of p21 by MPK38 (Figs. 1 and 2) are important for the regulation of p21 activity using diverse biochemical techniques and an adenoviral delivery system in diet-induced obese mice. Our findings imply that Thr55 phosphorylation of p21 by MPK38 may play a crucial role in the regulation of p21 activity. To verify this, we performed reporter assays to assess p21 promoter activity in the presence of MPK38, and found that MPK38 stimulates p21 promoter activity in a kinase-dependent manner (Fig. 4a). MPK38 also stimulated p21-mediated cell cycle arrest in a kinase-dependent manner, suggesting a potential role of MPK38 in adipocyte differentiation (Fig. 4e). Consistent with this, MPK38 kinase-dependently contributed to the p21-mediated metabolic regulation in obesity (Figs. 6 and 7).

p21 is known to be an unstable protein, having a half-life of about 30 min, and is degraded proteasomally in both an ubiquitin-dependent and an ubiquitin-independent manner^46,47^. Our results show that MPK38 inhibits Mdm2-dependent p21 ubiquitination *via* Th55 phosphorylation of p21 (Fig. 8c). Recent studies have also shown that p21 can be regulated by post-translational mechanisms^38^. For example, Ser146 phosphorylation by AKT/protein kinase B (PKB) stabilizes p21, whereas p21 is destabilized by glycogen synthase kinase (GSK)3β-mediated phosphorylation at Thr57^49,50^. However, Thr145 phosphorylation by AKT/PKB does not affect p21 stability but causes its cytoplasmic translocation^51^. Similarly, Ser153 phosphorylation by dual specificity tyrosine-phosphorylation-regulated kinase 1B (Dyrk1B) stimulates the translocation of p21 from the nucleus to the cytoplasm^52,53^. The present study has shown that MPK38 is capable of inducing greater stability and nuclear translocation of p21 through Thr55 phosphorylation. By contrast, the stability and subcellular localization of p21 are not affected by CDK and c-Jun N-terminal kinase (JNK)/p38-mediated Ser130 phosphorylation^54^.

Adipogenesis is tightly controlled by intricate transcription factor networks operating at different time points during differentiation^55,56^. PPARγ is considered a master regulator of adipogenesis from both in vitro and in vivo studies. Indeed, PPARγ is required for adipocyte differentiation^57,58^, and in many cases its expression is sufficient for the differentiation of non-adipose cells into adipocyte-like cells^59,60^. PPARγ also regulates insulin sensitivity, lipogenesis, and adipocyte survival and function^61^. Thus, it is reasonable proposition that p21, a transcriptional regulator, could regulate adipocyte differentiation by affecting transactivation by PPARγ. In the present study, we found that MPK38 plays a critical role in the association between p21 and PPARγ, following Thr55 phosphorylation of p21. Indeed, phosphorylated p21 strongly interacted with PPARγ in the nucleus, leading to inhibition of PPARγ binding to peroxisome proliferator response elements (PPRE) in target genes (Fig. 5d–f). This finding suggests a model in which p21 inhibits adipocyte differentiation by preventing PPARγ transcriptional activity as a result of a direct interaction with PPARγ in the nucleus (Fig. S7).

In conclusion, our findings demonstrate that MPK38 plays a key role in the positive regulation of p21 activity and function by phosphorylating p21 on Thr55, and suggest that MPK38 is a positive regulator of p21. However, further investigation of the effect of p21 phosphorylation at other sites directly related to its activity is necessary to clarify the molecular mechanisms of the regulation of obesity-associated metabolic changes by p21.

## Materials and methods

### Antibodies, plasmids, chemicals, MEF cells, oligonucleotides, and biochemical analyses

The antibodies, plasmids, and chemicals used have been described previously^8,42,62,63^. The anti-phospho-p21(T55) antibody was raised against the synthetic phosphopeptide FDFVTETPL, in which T represents phosphothreonine (Young In Frontier, Seoul, Korea), in a rabbit. The WWP-Luciferase (p21/WAF1 promoter) plasmid containing the p53-binding site was from Addgene (no. 16451). MEF^p21−/−^ cells were generated after timed matings of homozygous p21 and MEF^MPK38−/−^ has been described previously^42^. The oligonucleotides were from Bioneer Ltd (Cheongwon, Korea). Biochemical analyses, including co-immunoprecipitation, immunoblot analysis, luciferase assay, and in vitro kinase assay for MPK38, as well as quantitative real-time PCR (qPCR), confocal microscopy, and assays for apoptosis and cell cycle arrest, were performed using the indicated cells and experimental conditions, as previously described^2,8,42^.

### Construction of MPK38-mediated phosphorylation-defective p21 mutants

To achieve three p21 mutants (substitution of alanine for serine or threonine residues), wild-type p21 was used as the template for PCR with either the p21 forward or reverse primer (forward, 5’-GCGAATTCATGTCAGAACCGGCTGGG-3’; reverse, 5’-GCCTCGAGTTAGGGCTTCCTCTTGGA-3’; EcoRI/XhoI site underlined), together with one of the following pairs of primer sequences: for S116A, sense 5’-GTGGACCTGTCACTGGCTTGTACCCTTGTGCCT-3’, antisense 5’-AGGCACAAGGGTACAAGCCAGTGACAGGTCCAC-3’; for S153A, sense 5’-ACAGATTTCTACCACGCCAAACGCCGGCTGATC-3’, antisense 5’-GATCAGCCGGCGTTTGGCGTGGTAGAAATCTGT-3’; and for T55A, sense 5’-AACTTCGACTTTGTCGCCGAGACACCACTGGAG-3’, antisense 5’-CTCCAGTGGTGTCTCGGCGACAAAGTCGAAGTT-3’. The PCR products were cut with EcoRI and XhoI and then subcloned into pGEX4T-1 vector using EcoRI/XhoI site to generate pGEX4T-1-p21 substitution mutants.

### Generation of a p21 knock-in (T55A) cell line

Genome editing with the CRISPR/Cas9 system was performed in 3T3-L1 cells, as previously described^62,63^. Single-guide (sg) RNA (5’-AATGGCGGGCTGCATCCAGG-3’) was designed to target the genome adjacent to the p21 mutation site. The annealed oligonucleotides (5’-CACCGAATGGCGGGCTGCATCCAGG-3’ and 5’-AAACCCTGGATGCAGCCCGCCATTC-3’) containing the p21 guide sequence and Bbs1 ligation adapters were ligated into pX458 vector (Addgene) using 5 μl of 2 × Quick-Ligation Buffer and 1 μl of QuickLigase (New England BioLabs, Ipswich, MA). 3T3-L1 cells were co-transfected with 1 μg p21 sg RNA plasmid and pUC19 p21 T55A to generate the p21 knock-in (T55A) cell line. The clones obtained were then verified by DNA sequencing.

### Adipocyte differentiation and isolation of hepatocytes and adipocytes

3T3-L1 preadipocytes were transfected with the indicated plasmids using Amaxa Nucleofector technology (Amaxa, Cologne, Germany) according to manufacturers’ protocol (https://bioscience.lonza.com/lonza_bs/US/en/document/download/21296). The differentiation of 3T3-L1 cells was induced as previously described^64^. Briefly, 3T3-L1 cell differentiation was induced by treatment with differentiation medium (0.5 mM 3-isobutyl-1-methylzanthine (Sigma), 1 μM dexamethasone (Sigma), and 5 μg/ml insulin (Roche, Switzerland)) in Dulbecco’s modified Eagle’s medium (DMEM) containing 10% fetal bovine serum (FBS)) for 2 days. Culture medium was refreshed every 2 days with DMEM supplemented with 10% FBS and 5 μg/ml insulin. At day 10–14 of induction, fixed cells (4% formaldehyde, 1 h) were stained with the Oil Red O dye (Chemicon). Quantification of Oil Red O staining was achieved by measuring the absorbance at 520 nm. The isolation of hepatocytes and adipocytes was as previously described^42,62^.

### Animals and recombinant adenoviruses

All animal experiments were approved by the Chungbuk National University, and were conducted in accordance with the approved protocols and guidelines established by the Ethics Review Committee (CBNUA-966-16-02). Male C57BL/6 or p21-null mice (4-week-old) purchased from Jackson Laboratory were placed on a high-fat diet (HFD) (60% kcal fat; D12492, Research Diets, Inc.) for 8–10 weeks. They were kept under a 12 h light/dark cycle, with free access to autoclaved food and water, in specific pathogen-free (SPF) animal center. To prepare recombinant adenoviruses expressing wild-type (WT) and T55A p21, FLAG-tagged p21 plasmids (WT and T55A) were used as templates for PCR using the primers (5’-GTAACTATAACGGTCATGTCAGAACCGGCTGGGGATGTC-3’ and 5’-ATTACCTCTTTCTCCTTAGGGCTTCCTCTTGGAGAAGAT-3’) and the tail vein or epididymal fat pads of the HFD-fed mice were injected with adenovirus (approximately 1 × 10^9^ plaque-forming units), as previously described^9^.

### Metabolic analyses

Blood obtained from a tail vein was used to measure fed and fasted serum insulin and glucose in *ad libitum*-fed and 10 h-fasted HFD-fed mice infected with adenoviruses expressing GFP, WT p21, T55A p21, or MPK38 (positive control) using an ELISA kit (Crystal Chem) and an Accu-Check glucometer (Roche), respectively^42^. 2-deoxy-glucose uptake, ketone concentration, fatty acid oxidation rate, lipogenesis, and lipolysis were measured in HFD-fed mice infected with the indicated adenoviruses, as previously described^42,63,65^.

### Blood metabolic parameters

Blood samples were obtained from the abdominal aorta of 18 h-fasted HFD-fed mice infected with adenoviruses expressing GFP, WT p21, T55A p21, or MPK38. To separate the serum, whole blood samples were centrifuged at 2500 rpm at 4 °C for 15 min. An ELISA kit from R&D Systems (Joslin DERC Assay Core) was used to measure serum free fatty acid concentration. An automated serum analyzer (Hitachi 7080, Hitachi Science System Ltd, Ibaraki, Japan) was used to measure serum glucose, triglyceride, total cholesterol, HDL-cholesterol, and LDL-cholesterol. A triglyceride determination kit (Sigma) was used to measure serum triglyceride concentration. Serum concentrations of TNF-α, IL-6, IL-1β, and MCP1 were measured as previously described^63^.

### Glucose and insulin tolerance tests (GTTs/ITTs)

GTTs/ITTs were performed in fasted (~18 h for GTTs, ~6 h for ITTs) HFD-fed mice infected with the indicated adenoviruses. D-glucose (2 g/kg body mass) or insulin (0.75 U/kg mass in 0.1% bovine serum albumin (BSA); human R-insulin; Eli Lilly) was administrated intraperitoneally and blood glucose was measured from tail bleeds with an Accu-Check glucometer (Roche) at indicated time points after the administration.

### Statistical analysis

The data represent the means ± SEM of at least three independent experiments, and all experiments were performed in duplicate. Statistical analysis (GraphPad Prism software, version 6.0, La Jolla, CA) was performed using one-way or two-way ANOVA with Tukey’s multiple comparison test.

## Supplementary information

Supplementary Figure Legends

Supplementary Figure 1

Supplementary Figure 2

Supplementary Figure 3

Supplementary Figure 4

Supplementary Figure 5

Supplementary Figure 6

Supplementary Figure 7
