# Supplementary material for: Thr55 phosphorylation of p21 by MPK38/MELK ameliorates defects in glucose, lipid, and energy metabolism in diet-induced obese mice
Source: Cell Death Dis. 2019 May 16;10(6):380. doi: 10.1038/s41419-019-1616-z (PMC6522503; doi:10.1038/s41419-019-1616-z)
Supplement: Supplementary file 1 — Supplementary Figure Legends [file 41419_2019_1616_MOESM1_ESM.docx]

**Supplemental Information**

**Thr55 phosphorylation of p21 by MPK38/MELK ameliorates defects in glucose, lipid, and energy metabolism in diet-induced obese mice**

Running Title: Enhancement of p21 activity by MPK38

**Hyun-A Seong and Hyunjung Ha**

Department of Biochemistry, School of Biological Sciences, Chungbuk National University, Cheongju 28644, Republic of Korea

Address correspondence to:

**Hyunjung Ha**

Department of Biochemistry, School of Biological Sciences, Chungbuk National University, Cheongju 28644, Republic of Korea. Tel.: +82-43-261-3233; Fax: +82-43-267-2306; E-mail: [hyunha@cbnu.ac.kr](mailto:hyunha@cbnu.ac.kr)

**Supplementary Figure Legends**

**Fig. S1: Effect of Thr55 phosphorylation of p21 on the association of p53 with p300**

HEK293 cells transfected with the indicated expression vectors were lysed and then purified using glutathione-sepharose beads (GST Purification), and the extent of complex formation between p53 and p300 was determined by anti-FLAG antibody immunoblotting.

**Fig. S2: Effect of Thr55 phosphorylation of p21 on the mRNA expression of adipogenic genes in 3T3-L1 cells**

The relative mRNA expression of genes encoding key adipogenic proteins (*Ppar*γ, *Cebp*α, *Fabp4*, and *Adipoq*) in 3T3-L1 cells expressing p21 (WT or T55A) was quantified by densitometry, and the fold difference from control (vector) is presented. n = 6 per group, **p* < 0.05, ***p* < 0.01 compared to control. “Vector” indicates the use of empty vector alone.

**Fig. S3: Amelioration of adiposity and impaired glucose metabolism following Thr55 phosphorylation of p21 in HFD-fed p21^-/-^ mice**

**a** Size distribution of adipocytes (upper) and H&E-stained paraffin-embedded sections of epididymal WAT (lower) in HFD-fed p21^−/−^ mice infected with the indicated adenoviruses. MPK38 was used as a positive control. Scale bar, 100 μm. **b** Relative mRNA expression of adipogenic genes. Relative expression was quantified using densitometry, and the fold difference from control is presented. n = 6 per group, **p* < 0.05, ***p* < 0.01, ****p* < 0.001 compared to control. **c** Glucose tolerance tests were conducted by measuring blood glucose concentrations in mice following intraperitoneal injection of glucose (2 g/kg). n = 6 per group, **p* < 0.05, ***p* < 0.01, ****p* < 0.001 compared to control, determined by two-way ANOVA. **d** Insulin tolerance tests were performed by measuring blood glucose concentrations in mice following intraperitoneal injection of insulin (0.75 U/kg). n = 6 per group, **p* < 0.05, ***p* < 0.01, ****p* < 0.001 compared to control, determined by two-way ANOVA. **e, f** Blood glucose and serum insulin concentrations in fed and fasted (10 h) HFD-fed p21^−/−^ mice uninfected (control) or infected with the indicated adenoviruses. n = 6 per group, ***p* < 0.01, ****p* < 0.001 compared to fasted control, determined by two-way ANOVA. **g** *In vitro* ^3^H-2-deoxy-glucose uptake was measured in the presence or absence of human insulin (10 mU/ml) (left). n = 6 per group, **p* < 0.05, ***p* < 0.01 compared to insulin-treated control, determined by two-way ANOVA. IRS-PI3K signaling was evaluated using immunoblot analysis (right) after injection of insulin into the caudal vena cava (n = 3 per group)^42^. **h, i** Blood glucose (**h**) and relative mRNA expression of hepatic gluconeogenic genes (**i**). n = 6 per group, **p* < 0.05, ***p* < 0.01 compared to control.

**Fig. S4: HFD-fed mice infected with Ad-p21 have increased energy expenditure**

Oxygen consumption profiles (**a**), carbon dioxide generation profiles (**b**), RER profiles (**c**), energy expenditure (**d**), cumulative food intake (**e**), cumulative water intake (**f**), and locomotor activity (counts/1 h and total counts) (**g**) in HFD-fed C57BL/6 mice infected with the indicated adenoviruses during light and dark hours were measured using the OxyletPro™ System (PANLAB, Cornella, Spain). n = 6 per group, **p* < 0.05, ***p* < 0.01 compared to GFP. All data are represented as means ± SEM and analyzed by one-way ANOVA.

**Fig. S5: Effect of Thr55 phosphorylation of p21 on the mRNA expression of proinflammatory genes in HFD-fed** **C57BL/6 mice**

The mRNA expression of proinflammatory genes (*Tnf-α*, *Il-6*, *Il-1β*, and *Mcp1*) was measured using quantitative real-time PCR in WAT obtained from HFD-fed C57BL/6 male mice infected with the indicated adenoviruses. MPK38 was used as a positive control. Relative mRNA expression was quantified by densitometry, and the fold difference from control is presented. n = 6 per group, **p* < 0.05, ***p* < 0.01 compared to control.

**Fig. S6: Amelioration of abnormal lipid metabolism and inflammation following Thr55 phosphorylation of p21 in HFD-fed p21^-/-^ mice**

**a** Relative mRNA expression of lipogenic genes in liver and epididymal WAT, serum free fatty acid concentration, liver triglyceride content, and lipogenic capacity in hepatocytes. n = 6 per group, **p* < 0.05, ***p* < 0.01, ****p* < 0.001 compared to control. **b, c** Serum concentrations of total cholesterol (**b**), HDL-C (**b**), LDL-C (**b**), and proinflammatory proteins (**c**). **p* < 0.05, ***p* < 0.01, ****p* < 0.001 compared to control. **d** Relative mRNA expression of lipolytic genes in epididymal WAT. n = 6 per group, **p* < 0.05, ***p* < 0.01 compared to control. **e** Measurement of β-oxidation using ^14^C-labeled palmitate in liver. n = 6 per group, **p* < 0.05, ***p* < 0.01 compared to control. **f** Relative mRNA expression of fatty acid oxidative genes in epididymal WAT, serum triglyceride concentration, and the isoproterenol-stimulated lipolytic response of isolated adipocytes. n = 6 per group, **p* < 0.05, ***p* < 0.01, ****p* < 0.001 compared to control. **g** Representative images of H&E-stained sections of liver. n = 6 per group. Scale bar, 100 μm. **h, i** Total ketone body concentration in fed and fasted (18 h) serum (**h**) and the relative mRNA expression of genes involved in ketogenesis (*Ppar*α, *Cpt1*, and *Hmgcs2*) in liver (**i**). *Pck1* is a non-specific control. n = 6 per group, **p* < 0.05, ***p* < 0.01, ****p* < 0.001 compared to fasted control, determined by two-way ANOVA. **j** Phospho-S6 Ser240/244 levels in liver lysates from *ad libitum*-fed, fasted (18 h), and refed (2 h) HFD-fed p21^-/-^ mice uninfected or infected with the indicated adenoviruses (left). Immunoblot analyses of the mTORC1 signaling pathway in liver lysates (right). n = 6 per group.

**Fig. S7: A model for the role of Thr55 phosphorylation of p21 in PPARγ-mediated adipogenesis**

Activation of MPK38 by stress signals induces the Thr55 phosphorylation of p21 and the subsequent inhibition of PPARγ binding to PPREs of target genes. This eventually contributes to the suppression of adipogenesis by negatively regulating PPARγ transcriptional activity.
