# Supplementary figures and images for: Thr55 phosphorylation of p21 by MPK38/MELK ameliorates defects in glucose, lipid, and energy metabolism in diet-induced obese mice
Source: Cell Death Dis. 2019 May 16;10(6):380. doi: 10.1038/s41419-019-1616-z (PMC6522503; doi:10.1038/s41419-019-1616-z)

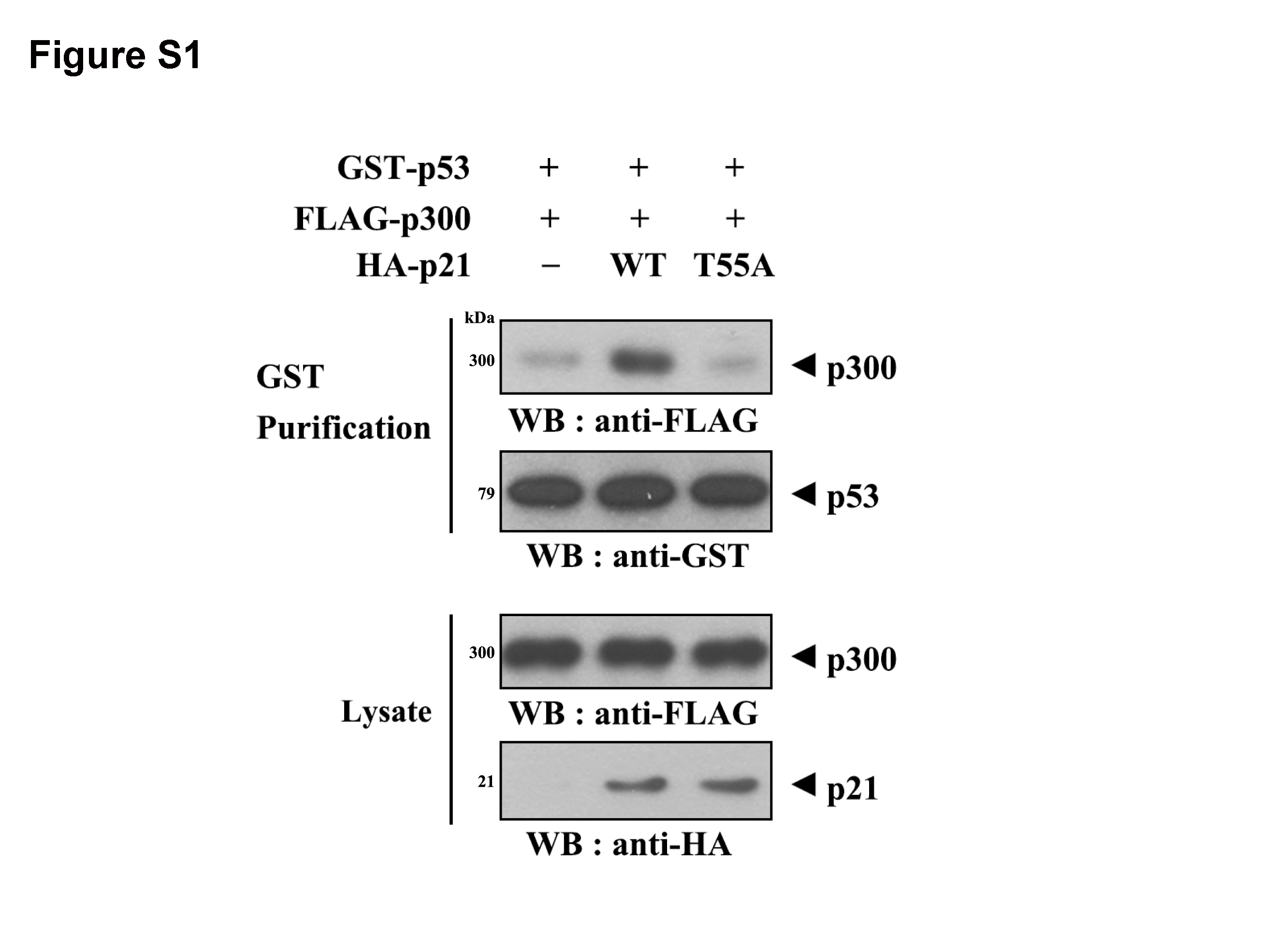

Supplement: Supplementary file 2 — Supplementary Figure 1 [file 41419_2019_1616_MOESM2_ESM.tif]

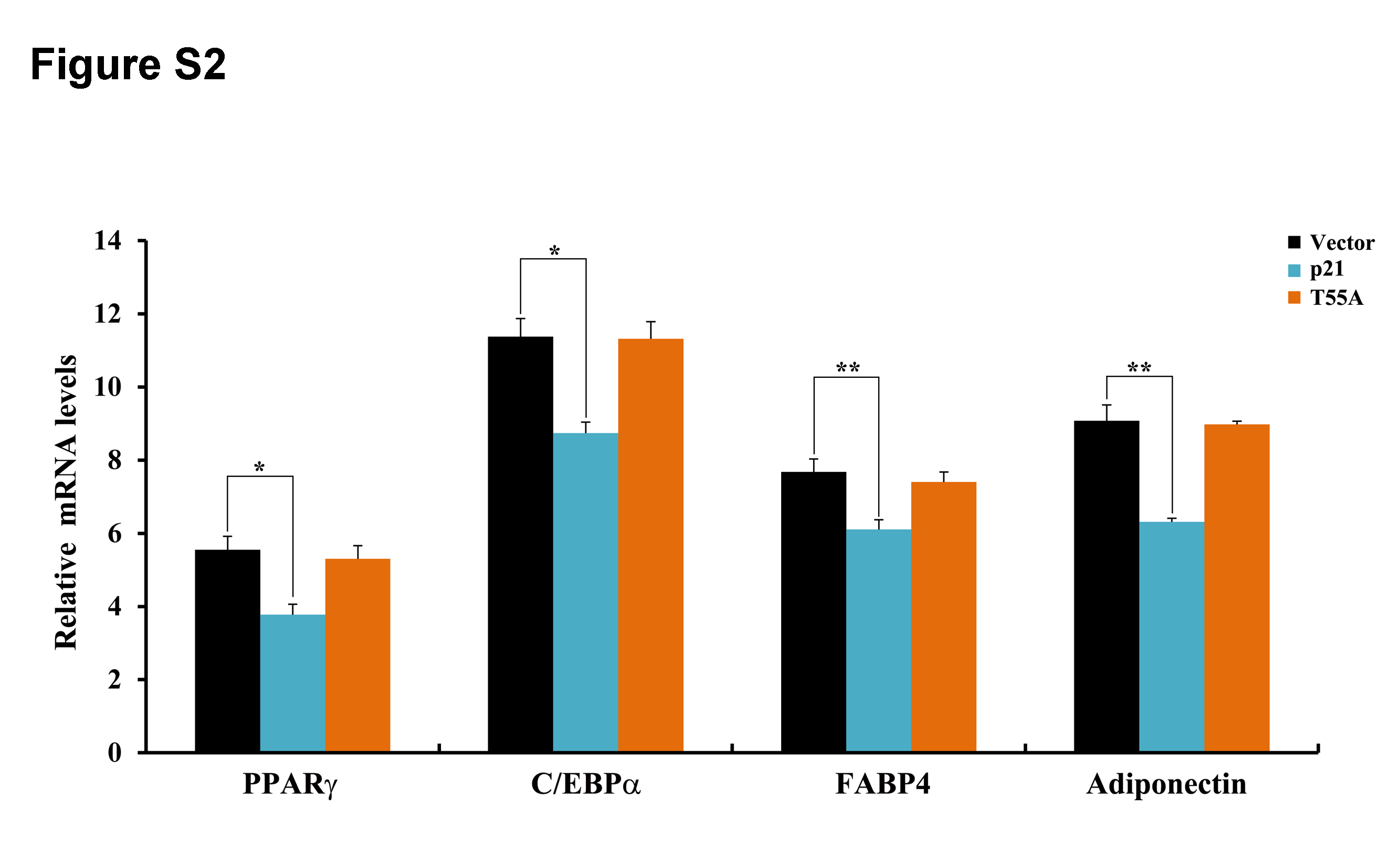

Supplement: Supplementary file 3 — Supplementary Figure 2 [file 41419_2019_1616_MOESM3_ESM.tif]

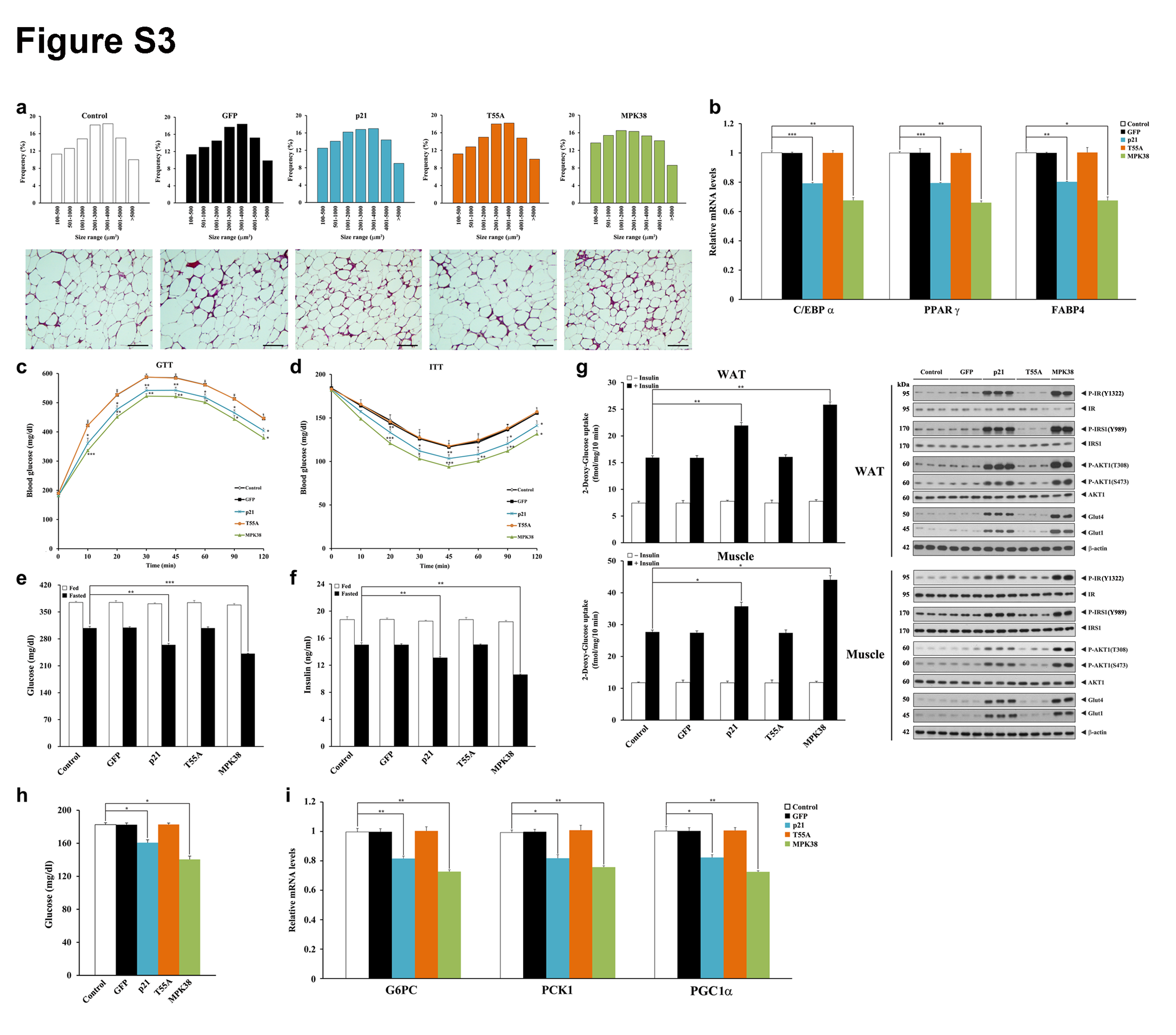

Supplement: Supplementary file 4 — Supplementary Figure 3 [file 41419_2019_1616_MOESM4_ESM.tif]

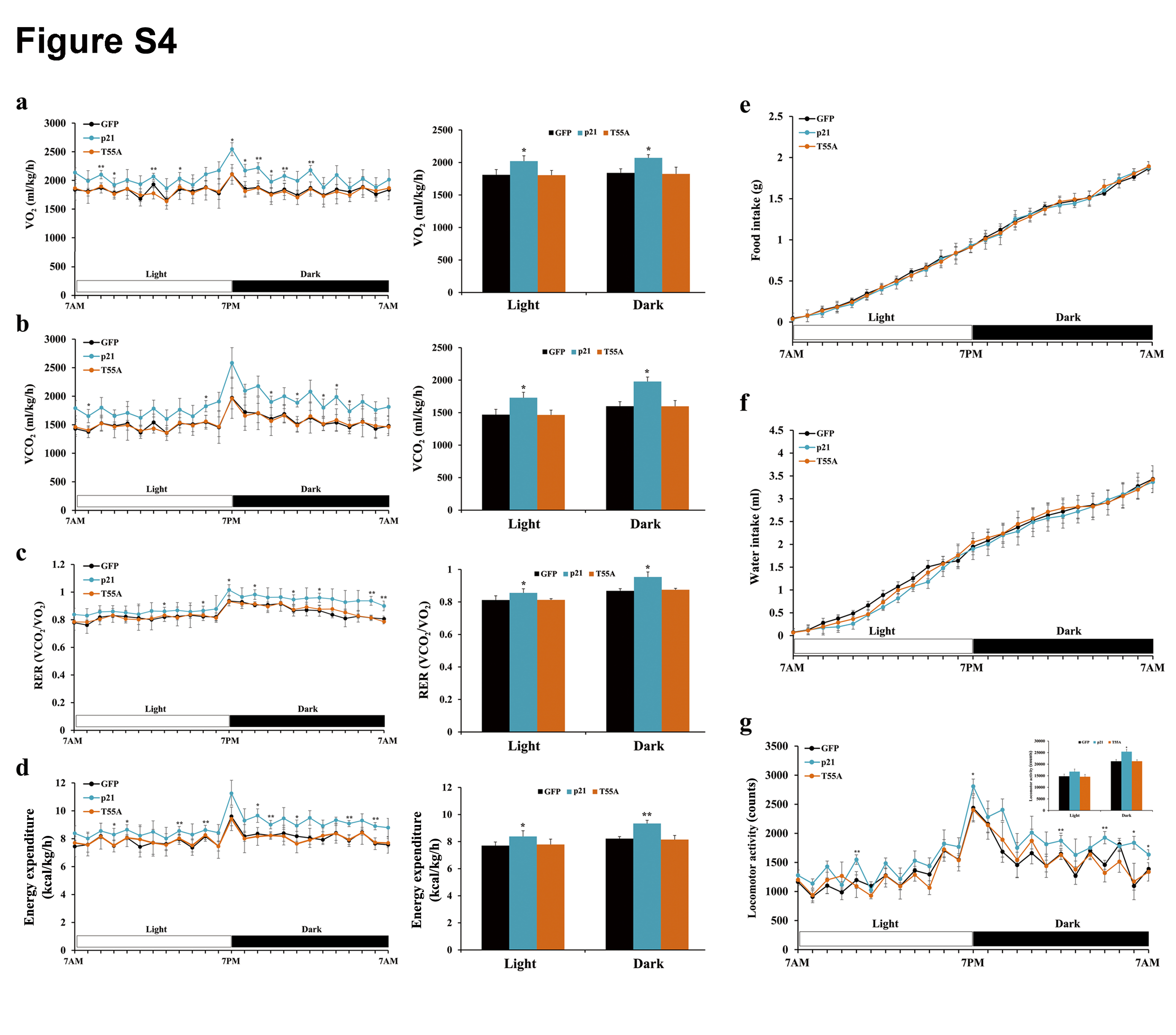

Supplement: Supplementary file 5 — Supplementary Figure 4 [file 41419_2019_1616_MOESM5_ESM.tif]

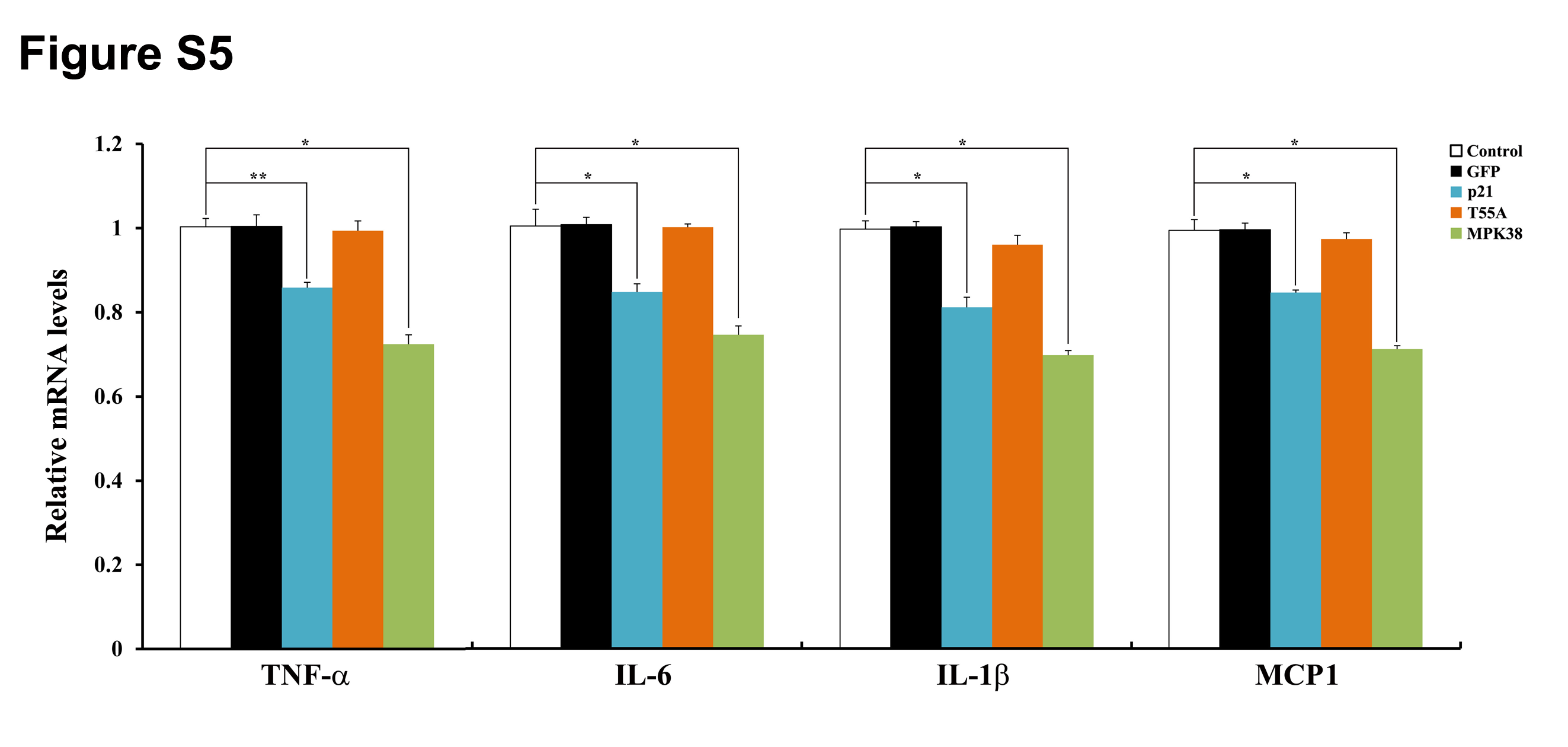

Supplement: Supplementary file 6 — Supplementary Figure 5 [file 41419_2019_1616_MOESM6_ESM.tif]

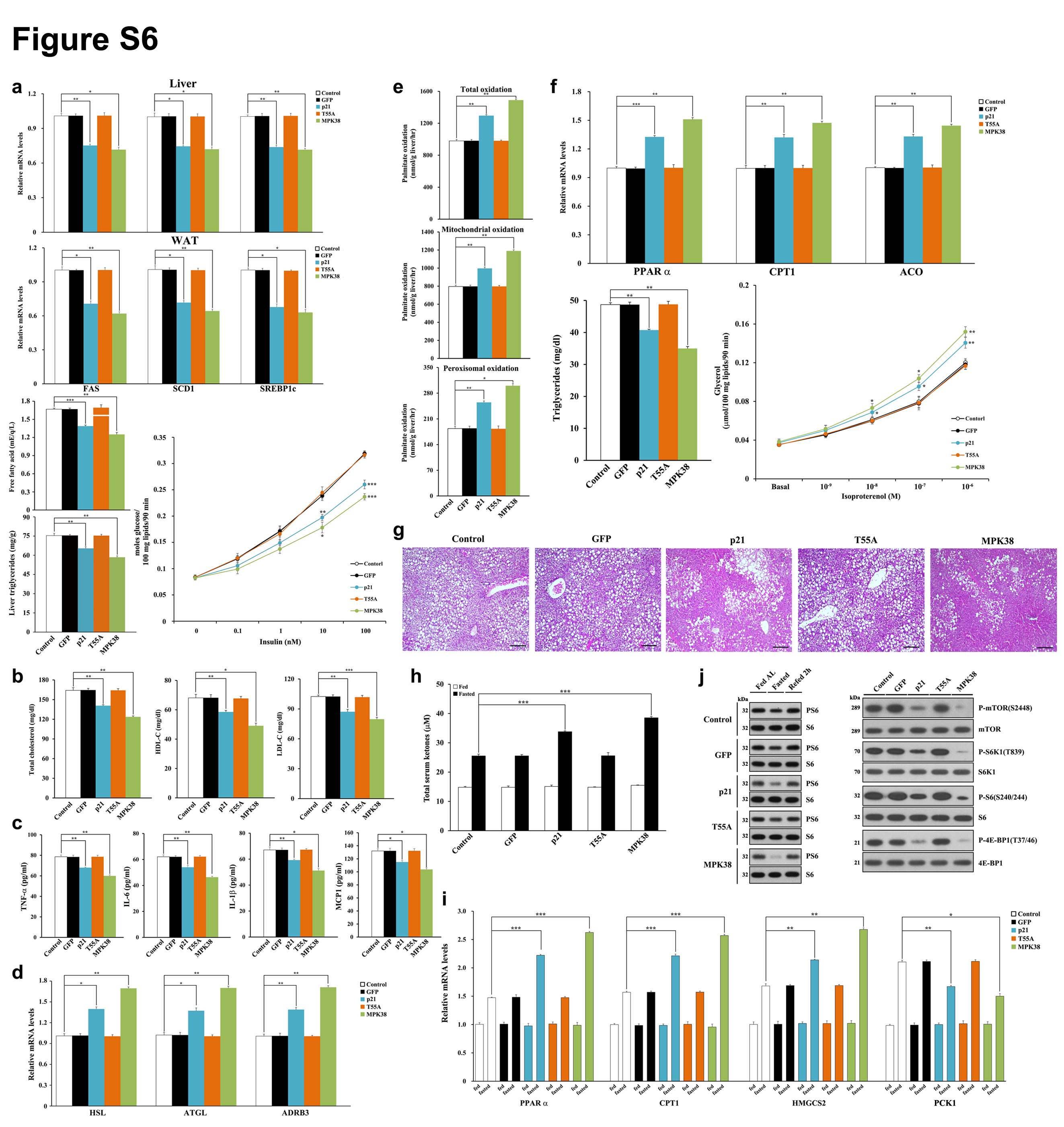

Supplement: Supplementary file 7 — Supplementary Figure 6 [file 41419_2019_1616_MOESM7_ESM.tif]

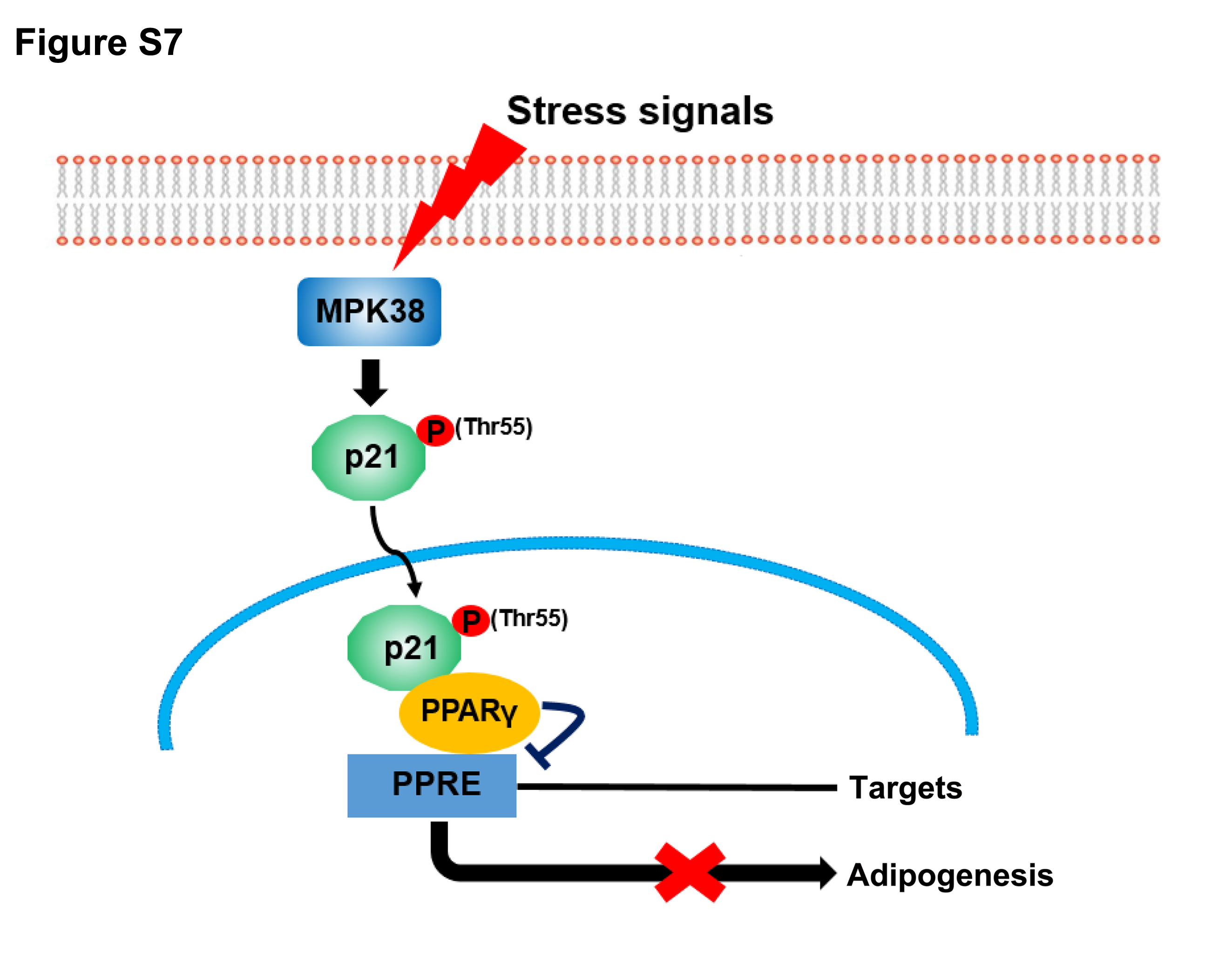

Supplement: Supplementary file 8 — Supplementary Figure 7 [file 41419_2019_1616_MOESM8_ESM.tif]
